# IL-7 coupled with IL-12 increases intratumoral T cell clonality, leading to complete regression of non-immunogenic tumors

**DOI:** 10.1007/s00262-021-02947-y

**Published:** 2021-04-28

**Authors:** Mamoru Tasaki, Midori Yamashita, Yukinori Arai, Takafumi Nakamura, Shinsuke Nakao

**Affiliations:** 1 Drug Discovery Research, Astellas Pharma Inc., 21, Miyukigaoka, Tsukuba, 305-8585 Japan; 2 Department of Biomedical Science, Graduate School of Medical Sciences, Tottori University, 86 Nishi-cho, Yonago, 683-8503 Japan

**Keywords:** IL-7, IL-12, Oncolytic virus, Intratumoral immune status, TCR repertoire

## Abstract

Immune checkpoint inhibitors against PD-1, PD-L1 and CTLA-4 have altered the treatment paradigm for various types of cancers in the past decade. However, they offer clinical benefits to only a subset of patients. Evaluation and identification of an appropriate therapeutic approach to improve intratumoral immune status are needed for better treatment outcomes. We previously demonstrated that intratumoral expression of IL-7 and IL-12 increased tumor-infiltrating lymphocytes in poorly immunogenic tumors, resulting in a higher tumor regression rate than IL-12 alone. However, the mechanism underlying the difference in efficacy with and without IL-7 remains unclear. Here, we identified a previously unknown effect of IL-7 on the T cell receptor (TCR) repertoire of intratumoral CD8^+^ T cells, which is induced in the presence of IL-12. While IL-7 alone increased the diversity of intratumoral CD8^+^ T cells, IL-7 with IL-12 increased a limited number of high-frequency clones, conversely augmenting IL-12 function to increase the clonality. The proportion of mice with multiple high-frequency clones in tumors correlated with that achieving complete tumor regression in efficacy studies. These findings provide a scientific rationale for combining IL-7 and IL-12 in anticancer immunotherapy and unveil a novel IL-7 function on intratumoral TCR repertoire.

**Supplementary information:**

The online version contains supplementary material available at 10.1007/s00262-021-02947-y.

## Introduction

Immune checkpoint blockade targeting PD-1/PD-L1 and CTLA-4 has markedly changed the paradigm of cancer therapy, providing durable clinical benefit to patients with various types of cancer [1–3]. However, the response rate remains low, likely due to the multidimensional and non-redundant immunosuppressive mechanisms in the tumor microenvironment [4, 5]. Therefore, optimal efficacy with successful changes in immune status in tumors is believed to require combination therapies using agents with different antitumor mechanisms [6, 7]. Effective changes in immune status involve an increase in tumor-infiltrating T cells as well as upregulation of inflammatory genes [6]. The most important response is the enrichment of polyclonal T cell clones with tumor-targeting properties, which may comprise the characteristic T cell receptor (TCR) repertoire observed in patients with clinical response [8–10]. Increased clonality of intratumoral T cells has been reported to be positively correlated with the clinical response of an anti-PD-1 antibody [11]. Sequential treatment with an anti-CTLA-4 antibody followed by an anti-PD-1 antibody increases the clonal T cell repertoire in tumors [12]. However, tumor-targeted radiotherapy, which may generate in situ vaccination following induction of immunogenic cell death of cancer cells, shows a broader TCR repertoire and increases antitumor efficacy to an anti-CTLA-4 antibody [13, 14]. Although these results appear contradictory, expansion of an increased but limited number of T cell clonotypes is likely to be associated with successful antitumor immunotherapies [10].

We have been studying in situ vaccination strategies using tumor-selective oncolytic vaccinia viruses carrying a series of immunomodulator(s), and previously demonstrated that intratumoral expression of IL-7 together with IL-12 contributed to a higher rate of complete regression (CR) of poorly immunogenic tumors [15]. This correlated with a higher number of infiltrating lymphocytes in tumors expressing both IL-7 and IL-12 than either cytokine alone. Considering that the difference in antitumor efficacy with and without IL-7 became apparent after a period of tumor suppression of one or two weeks, other immune phenotype changes, such as TCR repertoire, may have had a lasting effect on the degree of antitumor efficacy in the tumor microenvironment. Oncolytic virotherapy, as well as radiotherapy, may enhance the diversity of the TCR repertoire due to its in situ vaccination effect following release of multiple tumor antigens [16]. IL-12, a broad spectrum immune activator [17], and IL-7, a key molecule for T cell homeostasis [18], may also impact the architecture of the intratumoral TCR repertoire, though it is completely unknown whether IL-7, IL-12 and their combination in an oncolytic vaccinia virus platform enhance TCR diversity or clonality.

Here, using a poorly immunogenic LLC lung carcinoma model, we examined the activation status and diversity distribution of tumor-infiltrating T cells after intratumoral treatment with a series of recombinant oncolytic vaccinia viruses carrying transgenes to express IL-7, IL-12 or both, and investigated the association with antitumor efficacy. Whereas IL-12 alone increased clonality and IL-7 alone increased diversity, IL-7 together with IL-12 showed the exact opposite effect on intratumoral CD8^+^ T cells, markedly increasing clonality with expansion of a limited number of clonotypes in a higher proportion of mice, which positively correlated to the CR rate. Our data improve understanding of the dynamic role of IL-7 in the cytokine network, providing a scientific rationale for combined expression of IL-7 and IL-12 in antitumor therapies.

## Materials and methods

### Recombinant viruses

All recombinant vaccinia viruses used in this study were developed based on the attenuated vaccine strain, LC16mO, as described previously [15]. Briefly, LC16mO was modified with functional deletion of VGF and O1L and partial deletion of the B5R glycoprotein. DNA coding human IL-7 protein and DNA coding murine IL-12 protein were inserted into the VGF and O1L locus, respectively, using a plasmid containing the human IL-7 gene and a plasmid containing the murine IL-12 gene to generate hIL-7/mIL-12-VV. Similarly, a plasmid encoding human IL-7 protein and a plasmid encoding LacZ were used to generate hIL-7-VV; a plasmid encoding murine IL-12 protein and a plasmid encoding luciferase were used to generate mIL-12-VV; a plasmid encoding Discosoma sp. Red fluorescent protein (DsRed) and a plasmid encoding LacZ were used to generate Cont-VV. RK13 or A549 cells were used for virus propagation. Infected cells were collected, and viruses were purified by tangential flow filtration or density gradient ultracentrifugation using OptiPrep (Axis-Shield) according to the manufacturer’s protocol. Purified virus solution was titrated using a plaque assay in a monolayer culture of RK13 cells, in which the number of plaque-forming units (pfu) was determined. Virus solution was stored at  − 80 °C until use.

### Mice and cell lines

Male C57BL/6 mice purchased from Charles River Laboratories Japan, Inc. were maintained on a standard diet and water ad libitum throughout the experiments under specific pathogen-free conditions. Rabbit kidney RK13 cells, human lung carcinoma A549 cells and murine Lewis lung carcinoma (LLC) cells were purchased from American Type Culture Collection (ATCC). RK13 was cultured in Eagle’s minimum essential medium (ATCC) supplemented with 10% (v/v) heat-inactivated fetal bovine serum and 1% (v/v) penicillin–streptomycin (Thermo Fisher Scientific). A549 was cultured in F-12 K medium (ATCC) or Dulbecco’ s modified Eagle’s medium (DMEM) supplemented with 10% (v/v) heat-inactivated fetal bovine serum and 1% (v/v) penicillin–streptomycin. LLC was cultured in DMEM supplemented with 10% (v/v) heat-inactivated fetal bovine serum and 1% (v/v) penicillin–streptomycin. LLC-derived dendritic cells (DCs) were cultured in RPMI1640 (Sigma-Aldrich) supplemented with 10% (v/v) heat-inactivated fetal bovine serum and 1% (v/v) penicillin–streptomycin. Culture conditions were maintained in a humidified atmosphere with 5% CO_2_ at 37 °C. Cells were tested as mycoplasma-free.

### Syngeneic mouse model using Lewis lung carcinoma

Mice were subcutaneously inoculated with LLC cells into the right flank. Tumor diameter was measured using a digital caliper, and tumor size was calculated using the following formula: length × width^2^ × 0.52. After establishment of tumor burden, mice were randomly allocated to experimental groups such that mean tumor volume was similar among the groups, and intratumoral treatment with 30 μL of vehicle or virus suspension was started. Tumor size and body weight of individual mice were continuously monitored. Mice with signs of deterioration or acute weight loss were euthanized. Complete tumor regression was defined as complete tumor disappearance.

### Flow cytometry analysis

LLC tumors were dissected immediately after euthanasia by cervical dislocation under isoflurane anesthesia. Tumor tissues were minced with scissors and further mechanically dissociated using Tumor Dissociation Kit, mouse (Miltenyi Biotec) and GentleMACS (Miltenyi Biotec). Cells were passed through a nylon mesh filter and prepared for immunofluorescence staining and flow cytometry analysis. Spleens were minced with scissors and dissociated using BioMasher (Nippi, Japan), and red cells were subsequently lysed with ACK Lysing Buffer (Thermo Fisher Scientific). The following monoclonal antibodies were used for immunostaining: mouse CD4 PE-Cy7, mouse CD8a V500, mouse CD366 (Tim3) PE, mouse MHC Class II (I-A/I-E) PerCP-Cy5.5 (BD Biosciences); mouse CD127 (IL-7Rα) Brilliant Violet 510, mouse Interferon-γ PerCP-Cy5.5, mouse Granzyme B Alexa Fluor 647, mouse CD45 Alexa Fluor 647, mouse CD45R (B220) FITC, mouse CD11c PE, mouse CD103 PE-Cy7, mouse CD80 APC, and mouse CD279 (PD-1) PE-Cy7 (BioLegend). BD fixation/permeabilization kit (BD Biosciences) was used for intracellular staining. Zombie Aqua (BioLegend) or Fixable Viability Dye eFluor 780 (Thermo Fisher Scientific) was used to distinguish live and dead cells. Samples were acquired on MACSQuant Analyzer 10 (Miltenyi Biotec) and analyzed using FlowJo Ver.10 (BD Biosciences).

### NanoString gene expression analysis

LLC tumors (approximately 90 mm^3^) were treated with 30 μL of PBS or virus suspension every other day for a total three times. Three days after the last treatment, tumor samples were collected in ISOGEN (Nippongene) immediately after euthanasia by cervical dislocation under isoflurane anesthesia. Total RNA was extracted using linear acrylamide and isopropanol, hybridized with the PanCancer Mouse Immune Profiling panel (NanoString Technologies) and analyzed using the nCounter DX Analysis System (NanoString). Data were processed using nSolver Analysis Software and the Advanced Analysis module (NanoString).

### TCR repertoire analysis

LLC tumors in the right flank of mice were intratumorally treated with 30 μL of PBS or virus suspension every other day for a total of three times. Eleven or twelve days after the last treatment, spleens and tumors were dissected. Spleens were minced with scissors and further mechanically dissociated using Spleen Dissociation Kit, mouse (Miltenyi Biotec) and GentleMACS. After removal of dead cells using the Dead Cell Removal Kit (Miltenyi Biotec), splenic CD4^+^ and CD8^+^ T cells were isolated using CD4^+^ T Cell Isolation kit, mouse (Miltenyi Biotec), and CD8a^+^ T Cell Isolation Kit, mouse (Miltenyi Biotec), respectively, according to the manufacturer’s protocols. Likewise, tumors were dissociated using Tumor Dissociation kit, mouse and dead cells were removed. Intratumoral CD4^+^ and CD8^+^ T cells were isolated using CD4 (TIL) MicroBeads, mouse (Miltenyi Biotec) and CD8a^+^ T Cell Isolation Kit, mouse, respectively. Isolated immune cells were collected in ISOGEN. RNA extracted using the RNeasy Plus Universal Mini kit (QIAGEN) was reverse transcribed into complementary DNA (cDNA) using SuperScript III Reverse Transcriptase (Thermo Fisher Scientific). Subsequently, double-stranded cDNA was synthesized, and TCR-*β*-chain genes were amplified using adaptor ligation-mediated PCR [19]. High-throughput sequencing was performed using MiSeq Reagent Kit v3 (600 Cycle) (Illumina, San Diego, CA, USA) and MiSeq (Illumina). Reads with a quality value (QV) score ≥ 20 were used for analyses [20]. TRBV and TRBJ segments in TCR genes were assigned based on the international ImMunoGeneTics information system (IMGT) database. A unique sequence read was defined as a sequence read having no identity in TRBV, TRBJ and deduced amino acid sequence of TCR-β-chain CDR3 with the other sequence reads. Data processing including calculation of the inverse Simpson's diversity index was performed using a repertoire analysis software (Repertoire Genesis, Osaka, Japan).

### Tumor rechallenge study

C57BL/6 mice that had been previously cured of subcutaneous LLC tumors by treatment with a mixture of hIL-7-VV and mIL-12-VV were subcutaneously inoculated with 4 × 10^5^ LLC cells 74 days after the last viral treatment. Growth of rechallenged tumors was continuously monitored. Age-matched treatment-naïve mice were used as a control group.

### Real-time PCR

For RNA analysis of *Cxcl3*, *Ccl3* and *Clec4n* in LLC-derived DCs, CD11c^+^ cells were isolated from mIL-12-VV-treated LLC tumors using EasySep Mouse CD11c Positive Selection Kit II (STEMCELL Technologies). Cells were plated at 5 × 10^4^ cells per well in a 96-well plate with serially diluted recombinant human IL-7 (PEPROTECH) or 1 μg/mL LPS (Sigma-Aldrich) and incubated for 5 h. Subsequently, total RNA was extracted using the RNeasy Plus Micro Kit (QIAGEN), and RNA was converted to cDNA using SuperScript IV VILO Master Mix (Thermo Fisher Scientific). Real-time PCR was performed using the TaqMan Gene Expression Master Mix and specific primers and probes from TaqMan Gene Expression Assays (Thermo Fisher Scientific). The mRNA expression level of each gene relative to *β-actin* was calculated using the ΔΔCt method.

### Statistical analysis

Statistical analysis was conducted using GraphPad Prism 8 (GraphPad Software, San Diego CA, USA). Procedures for comparison are described in each figure. *P* values < 0.05 were considered significant.

## Results

### IL-7 together with IL-12 increased infiltration of activated T cells in poorly immunogenic tumors

**Fig. 1:**
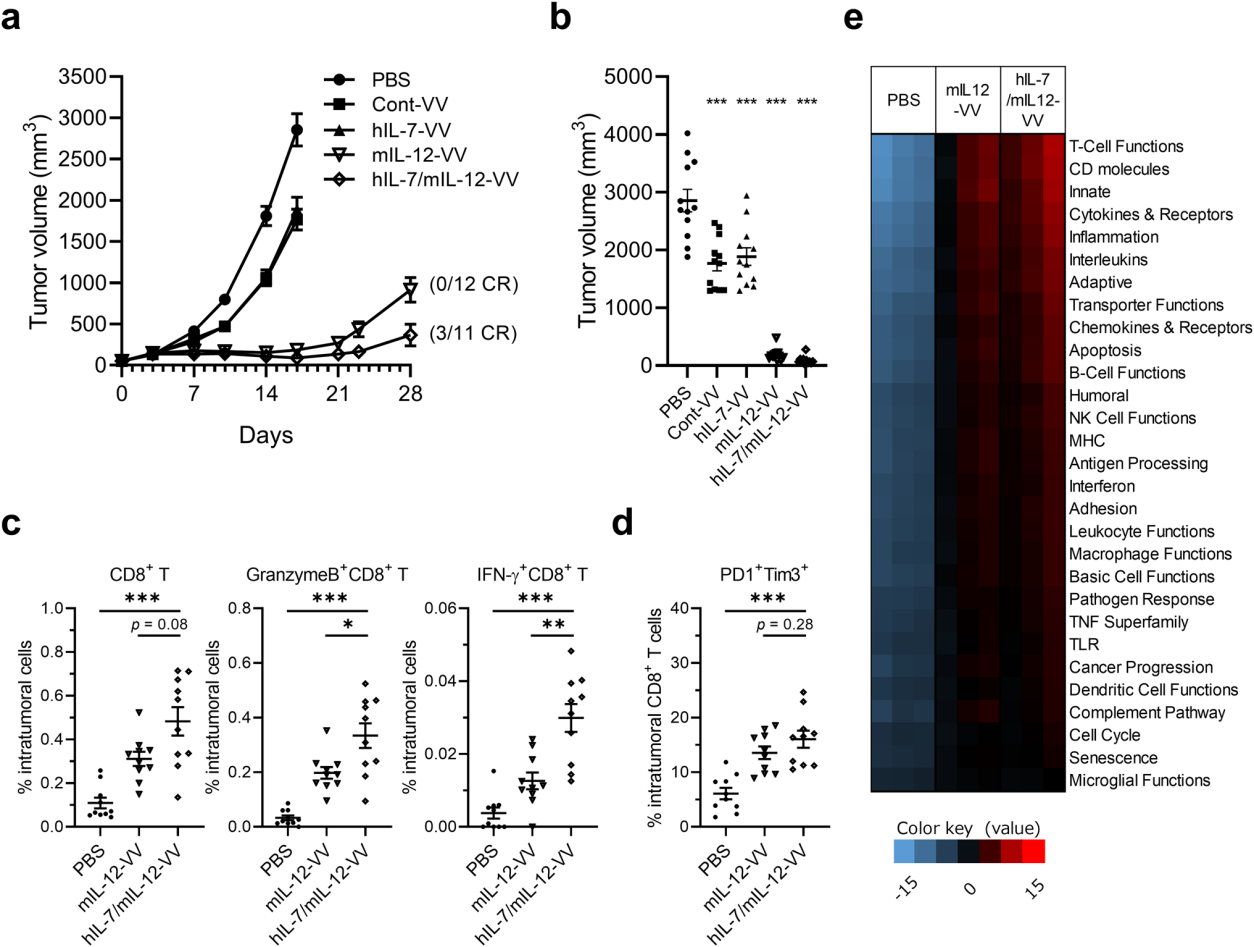
Improved antitumor efficacy and increased infiltration of activated T cells in tumors after intratumoral expression of IL-7 in addition to IL-12. **a**, **b** Mice bearing subcutaneous LLC tumors were intratumorally injected with PBS, Cont-VV, hIL-7-VV, mIL-12-VV or hIL-7/mIL-12-VV (2 × 10^7^ pfu) on Days 1, 3 and 5 (*n* = 11 to 12). **a** LLC tumor growth is shown with the proportion of mice that achieved complete tumor regression (CR). **b** Tumor volume on Day 17. ***p < 0.001 versus PBS by Dunnett’s multiple comparisons test. **c**, **d** Mice bearing subcutaneous LLC tumors were treated with a single injection of PBS, mIL-12-VV or hIL-7/mIL-12-VV (2 × 10^7^ pfu). Six days later, tumor-infiltrating CD8^+^ T cells were analyzed by flow cytometry (*n* = 10 per group). *p < 0.05, **p < 0.01, and ***p < 0.001 by Mann–Whitney *U* test. **e** LLC-bearing mice were treated with PBS, mIL-12-VV or hIL-7/mIL-12-VV (2 × 10^7^ pfu) every other day for a total of three times (*n* = 3). Three days after the last treatment, tumors were dissected. Total RNA was isolated and subjected to gene expression analysis using the NanoString PanCancer immune panel. Red indicates high expression, and blue indicates low expression. Data with bars indicate mean ± SEM

First, we evaluated the antitumor activity of a recombinant oncolytic vaccinia virus carrying transgenes to express human IL-7 and murine IL-12 (hIL-7/mIL-12-VV) and compared it to a virus expressing human IL-7 (hIL-7-VV; human IL-7 is reactive for mouse immune cells [21]), murine IL-12 (mIL-12-VV) or neither cytokine (Cont-VV) (Supplementary Fig 1), against LLC tumors, which are known to be poorly immunogenic [22, 23]. Significant antitumor efficacy was observed in all viral treatment groups (*p* < 0.001 for all groups) on Day 17, with three of eleven mice treated with hIL-7/mIL-12-VV achieving CR on Day 28 compared to none in the other groups (Fig. 1a, b), which is consistent with our previous study [15]. Next, we examined the activation status of intratumoral immune cells. Whereas the number of total CD8^+^ T cells tended to be higher in tumors treated with hIL-7/mIL-12-VV than mIL-12-VV, the number of activated CD8^+^ T cells expressing granzyme B or IFNγ was significantly higher in tumors treated with hIL-7/mIL-12-VV (Fig. 1c, Supplementary Fig 2). There was no apparent difference in the number of PD-1^+^Tim3^+^ exhausted CD8^+^ T cells between tumors treated with hIL-7/mIL-12-VV and mIL-12-VV (Fig. 1d, Supplementary Fig 2). Furthermore, we performed multiplexed gene expression analysis of tumors treated with PBS, mIL-12-VV or hIL-7/mIL-12-VV using the NanoString nCounter system (NanoString Technologies). Treatment with hIL-7/mIL-12-VV upregulated various immune pathways, which were similarly upregulated following treatment with mIL-12-VV (Fig. 1e). Direct comparison of hIL-7/mIL-12-VV-treated tumors and mIL-12-VV-treated tumors showed significant changes in gene expression related to antigen-presenting cells (APCs): *Clec4n*, a C-type lectin receptor on dendritic cells (DCs) [24], and *Cxcl3* and *Ccl3*, chemokines released from DCs and macrophages [25, 26] were upregulated, while *Axl*, a receptor tyrosine kinase which negatively regulates DCs to result in T cell inhibition [27], was downregulated in hIL-7/mIL-12-VV-treated tumors (Table 1). Little to no IL-7Rα expression was observed in intratumoral DCs regardless of the presence or absence of IL-12. Further, no upregulation of *Cxcl3*, *Ccl3* or *Clec4n* was observed in isolated DCs following stimulation with IL-7 (Supplementary Fig 3), suggesting that IL-7 does not act directly on DCs to upregulate these genes. The number of tumor-infiltrating DCs, mature DCs expressing major histocompatibility complex (MHC) class II and activated CD103^+^ DCs with high expression of CD80 was identical between hIL-7/mIL-12-VV- and mIL-12-VV-treated tumors (Supplementary Fig 4).

**Table 1:** Genes upregulated by hIL-7/mIL-12-VV compared to mIL-12-VV

Gene	Log2-fold change	Lower confidence limit (log2)	Upper confidence limit (log2)	P-value
*Cxcl3*	2.01	1.66	2.37	0.000366
*S100a8*	1.58	1.19	1.97	0.00138
*Slc7a11*	1.04	0.723	1.35	0.00293
*Ifitm1*	0.919	0.619	1.22	0.00389
*Ccl3*	1.32	0.839	1.8	0.00573
*Cxcl2*	1.14	0.676	1.59	0.00839
*Msln*	− 0.832	− 1.17	− 0.491	0.00876
*Klrc1*	1.56	0.908	2.22	0.00944
*Axl*	− 0.478	− 0.684	− 0.271	0.0106
*Igf1r*	− 0.44	− 0.63	− 0.25	0.0106
*Clec4n*	1.15	0.648	1.65	0.0109
*Nt5e*	0.466	0.286	0.645	0.0147
*Il7r*	1.22	0.627	1.81	0.0156
*Arg1*	0.684	0.318	1.05	0.0215
*Col4a1*	− 0.441	− 0.697	− 0.185	0.0279
*Il1rn*	0.977	0.397	1.56	0.0299
*Anxa1*	− 0.355	− 0.566	− 0.144	0.0301
*Cd14*	0.674	0.271	1.08	0.0306
*Dock9*	− 0.514	− 0.834	− 0.195	0.0344
*Klrd1*	1.28	0.465	2.1	0.037
*Cd96*	0.621	0.245	0.996	0.0479
*Havcr2*	0.741	0.222	1.26	0.0489
*Il1b*	0.72	0.216	1.22	0.0489

### IL-7 shows opposite effects in facilitating clonality of intratumoral CD8^+^ T cells with and without IL-12

**Fig. 2:**
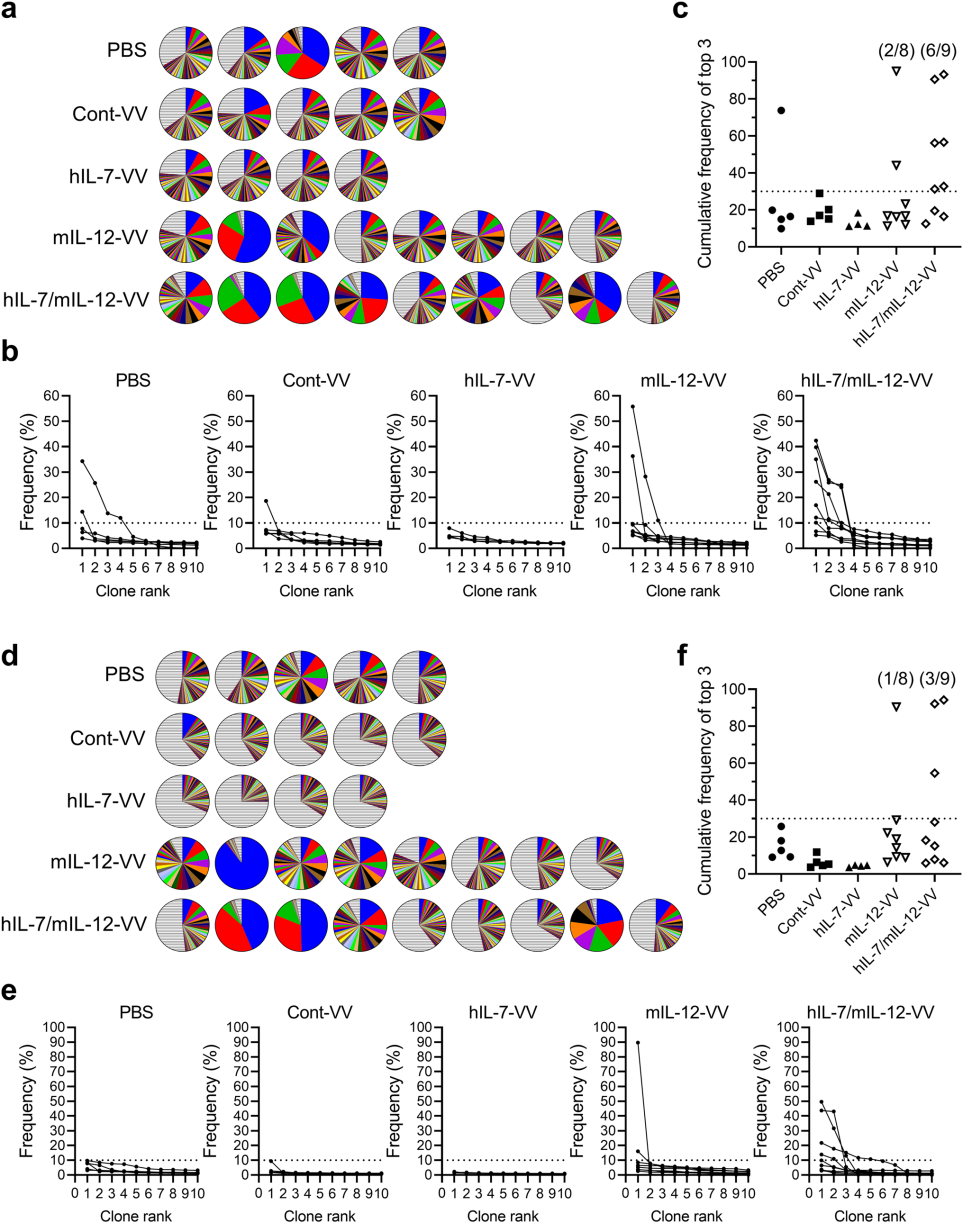
Intratumoral expression of IL-7 in addition to IL-12 increases the number of high-frequency T cell clones in tumors. Mice bearing subcutaneous LLC tumors were injected with PBS, Cont-VV, hIL-7-VV, mIL-12-VV or hIL-7/mIL-12-VV (2 × 10^7^ pfu) every other day for a total of three times. Eleven or twelve days after the last treatment, intratumoral CD8^+^ and CD4^+^ T cells were sorted, and CDR3 sequences were analyzed as described in the Methods. **a** Pie charts showing the frequency of CD8^+^ T cell clones as a percentage of all clones in tumors from individual mice. Colored areas indicate the top 50 frequent clones in each sample. Striped areas represent the sum of the remaining clones. The colors do not correspond to the same T cell clones among pie charts. **b** Frequency of the top 10 CD8^+^ T cell clones in tumors. **c** Cumulative frequency of the top 3 CD8^+^ T cell clones in each treatment group. **d** Pie charts showing the frequency of CD4^+^ T cell clones as a percentage of all clones in tumors from individual mice. **e** Frequency of the top 10 CD4^+^ T cell clones in tumors. **f** Cumulative frequency of the top 3 CD4^+^ T cell clones in each treatment group

**Fig. 3:**
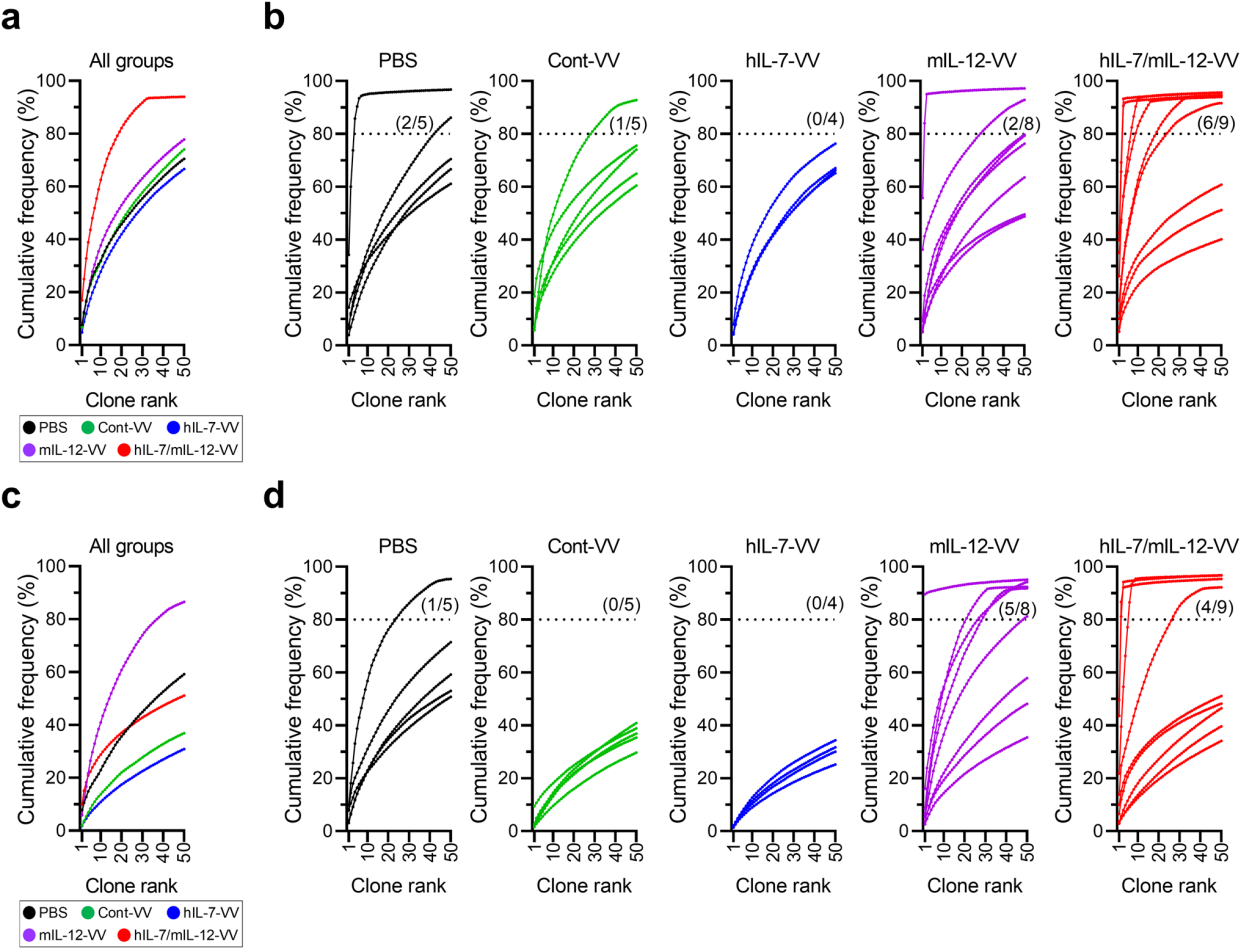
Characteristics of the TCR repertoire frequency distribution in tumor. The top 50 intratumoral T cell clones are ordered according to frequency on the x-axis and the cumulative percentage of total in-frame reads is plotted on the y-axis. **a, c** The group median frequency was calculated for each clone rank in intratumoral CD8^+^ T cells (**a**) and CD4^+^ T cells (**c**). **b, d** Frequency distribution of CD8^+^ T cells (**b**) and CD4^+^ T cells (**d**) in individual mice in each treatment group. *n* = 5 for PBS and Cont-VV; *n* = 4 for hIL-7-VV; *n* = 8 for mIL-12-VV; *n* = 9 for hIL-7/mIL-12-VV. Dotted line indicates the 80% border of the repertoire space

Despite the minor molecular differences related to APCs between tumors treated with hIL-7/mIL-12-VV and mIL-12-VV, we hypothesized that this difference may have a lasting effect on intratumoral T cells, resulting in differences in antitumor efficacy. We isolated CD8^+^ and CD4^+^ T cells from tumors treated with PBS, Cont-VV, hIL-7-VV, mIL-12-VV or hIL-7/mIL-12-VV, sequenced the complementarity determining region (CDR) 3 of TCRβ, calculated the frequency of unique clones in each sample and ranked the top 50 clones from highest to lowest frequency (Fig. 2a, d). With respect to intratumoral CD8^+^ T cells, high-frequency (> 10%) clones were observed in seven out of nine mice treated with hIL-7/mIL-12-VV, compared to a lower proportion of mice treated with PBS, Cont-VV, hIL-7-VV and mIL-12-VV (two of five, one of five, none of four, and two of eight, respectively) (Fig. 2b). Furthermore, five out of nine mice treated with hIL-7/mIL-12-VV had multiple high-frequency clones, compared to only one out of eight mice treated with mIL-12-VV (Fig. 2b), resulting in higher cumulative frequencies of the top 3 clones in hIL-7/mIL-12-VV-treated mice than those in mIL-12-VV-treated mice (Fig. 2c). Likewise, high-frequency CD4^+^ T cell clones were observed in four out of nine mice treated with hIL-7/mIL-12-VV, compared to only two out of eight mice treated with mIL-12-VV and no mice treated with PBS, Cont-VV or hIL-7-VV (Fig. 2e). Four out of nine mice treated with hIL-7/mIL-12-VV, compared to no mice in the other experimental groups, had multiple high-frequency clones (Fig. 2e). Cumulative frequencies of the top 3 CD4^+^ T cell clones in hIL-7/mIL-12-VV-treated mice were expectedly higher than those in mice in the other groups (Fig. 2f). Next, to analyze the overall clonal distribution, we plotted the cumulative frequencies of the top 50 clones in each animal. The group median distribution of intratumoral CD8^+^ T cell clones revealed that Cont-VV induced minor changes, while hIL-7-VV comparatively increased diversity and mIL-12-VV increased clonality (Fig. 3a). Strikingly, hIL-7/mIL-12-VV increased the clonality of intratumoral CD8^+^ T cells and a higher proportion of mice (six out of nine) showed cumulative frequencies greater than 80% for the top 50 clones compared to the other groups, including mIL-12-VV (Fig. 3b). Changes in intratumoral CD8^+^ T cell diversity were also supported by trends observed in the inverse Simpson's diversity index (Supplementary Fig. 5). The effect of these viruses on intratumoral CD4^+^ T cell clonality differed from that on CD8^+^ T cells: Cont-VV and hIL-7-VV increased diversity, while mIL-12-VV most markedly increased clonality compared to hIL-7/mIL-12-VV (Fig. 3c, d, Supplementary Fig. 6). These results indicate completely different effects of IL-7 on CD8^+^ T cell diversity with and without IL-12.

### Local expression of IL-7 and IL-12 systemically affects TCR repertoire

**Fig. 4:**
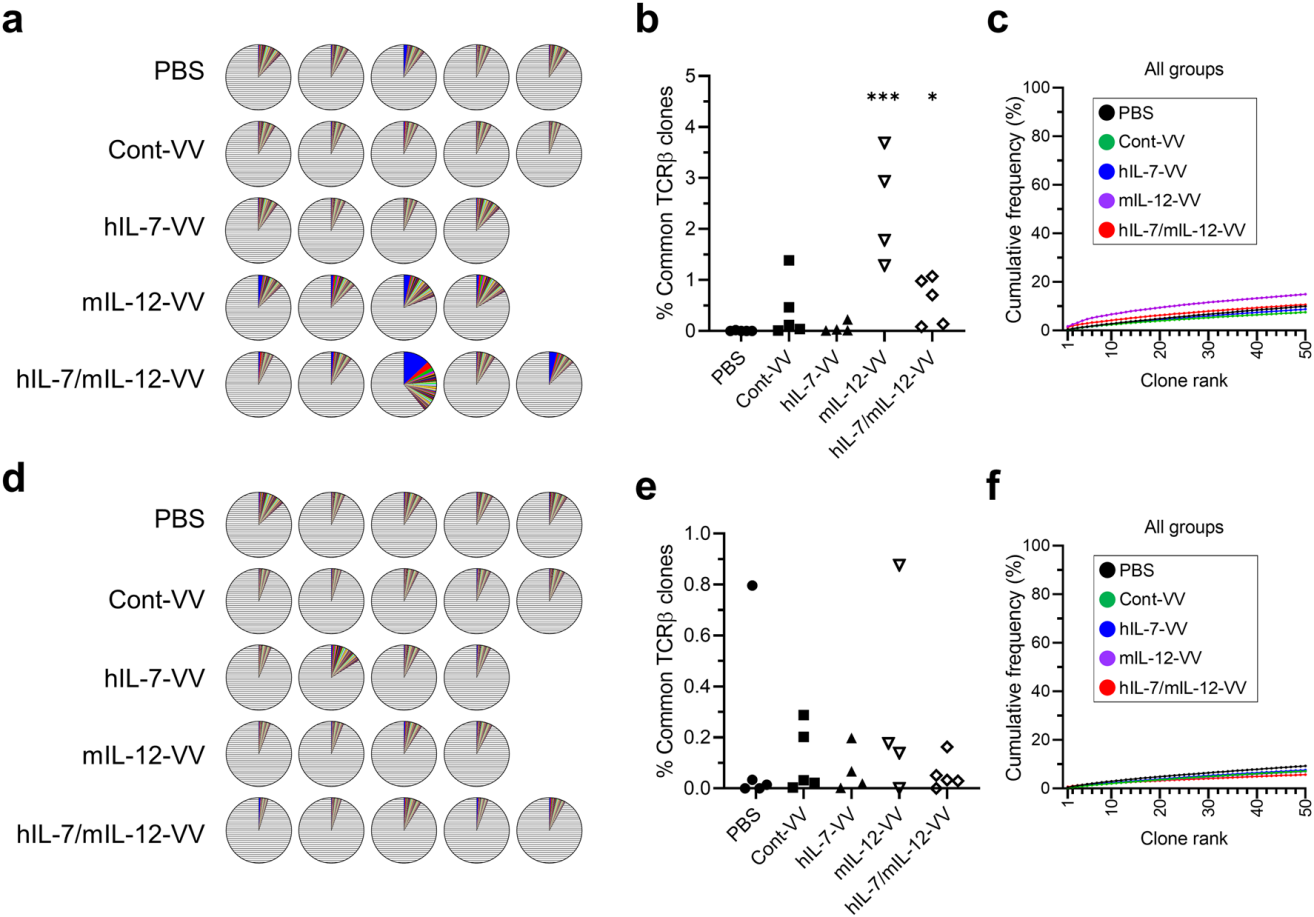
T cell diversity in spleens. Spleens were obtained from LLC-bearing mice intratumorally treated with PBS, Cont-VV, hIL-7-VV, mIL-12-VV or hIL-7/mIL-12-VV as described in Fig. 2, at the time of tumor collection. Splenic CD8^+^ and CD4^+^ T cells were sorted, and CDR3 sequences were analyzed as described in the Methods. **a, d** Pie charts showing the frequency of CD8^+^ T cell clones (**a**) and CD4^+^ T cell clones (**d**) as a percentage of all clones in the respective cell types in the spleen of individual mice. **b, e** Cumulative percentage of splenic CD8^+^ T cell clones (**b**) and CD4^+^ T cell clones (**e**) identified among the top 5 clones in tumors. *p < 0.05 and ***p < 0.001 versus PBS by Dunn’s multiple comparisons test. **c, f** Top 50 splenic CD8^+^ T cell clones (**c**) and CD4^+^ T cell clones (**f**) are ordered according to frequency on the x-axis and the cumulative percentage of total in-frame reads is plotted on the y-axis. The group median frequency was calculated for each clone rank. *n* = 5 for PBS, Cont-VV and hIL-7/mIL-12-VV; *n* = 4 for hIL-7-VV and mIL-12-VV

We previously reported that intratumoral expression of IL-7 and IL-12 mediated by an oncolytic vaccinia virus led to the establishment of antitumor memory, resulting in rejection of rechallenged tumors [15]. In this study, we again demonstrated that mice treated with hIL-7-VV in combination with mIL-12-VV more than 90 days prior rejected the rechallenged LLC tumors (Supplementary Fig. 7), suggesting that T cell clones reactive to LLC tumors may migrate to, and be stored in, secondary organs. We examined splenic T cells and assessed whether changes in the intratumoral TCR repertoire after viral treatment systemically affects T cell clonality. The diversity of the TCR repertoire of CD8^+^ and CD4^+^ T cells in the spleen was much higher than in tumors (Fig. 4a, d). Compared to observing little to no CD8^+^ T cell clones that were identified in tumors in the spleen of mice treated with PBS, we found T cell clones with the same CDR3 sequence in the spleen of animals treated with Cont-VV, hIL-7-VV, mIL-12-VV and hIL-7/mIL-12-VV, indicating migration of intratumoral CD8^+^ T cells into secondary lymphoid organs, as expected (Table 2). The high cumulative frequency of the top 5 splenic clones in mIL-12-VV-treated and hIL-7/mIL-12-VV-treated mice (Fig. 4b) is consistent with the stronger antitumor efficacy of mIL-12-VV and hIL-7/mIL-12-VV compared to Cont-VV or hIL-7-VV. However, the cumulative frequency and overall clonality calculated for the top 50 clones were higher in mIL-12-VV-treated mice than hIL-7/mIL-12-VV-treated mice, which is in contrast to the proportion of mice that achieved CR (Fig. 4c). High-frequency T cell clones found in the spleen were not always highly frequent in tumors in the treatment groups. Furthermore, some T cell clones that were identified among the top 3 high-frequency clones in tumors were absent from the spleen. Unlike CD8^+^ T cells, there were no obvious differences in the cumulative frequency of the top 5 clones or the overall clonality of CD4^+^ T cells in the spleen among treatment groups (Fig. 4e, f, and Supplementary Table 1).

**Table 2:** Frequency of top 5 intratumoral CD8^+^ T cell clones in the spleen

Mouse	CDR3β sequence	% in tumor	% in spleen
PBS-1	CGARQSNTEVFF	3.93	0.00
	CASSLDWGDYEQYF	3.02	0.00
	CASRQGEQYF	2.91	0.00
	CASSQVLGDTQYF	2.76	0.00
	CASSPDWVYAEQFF	2.50	0.00
PBS-2	CASSLELGGPTQYF	14.41	0.00
	CASSQEQTINYAEQFF	3.15	0.00
	CASSIKVANTEVFF	2.29	0.00
	CASSLLGAYEQYF	2.26	0.02
	CASSPTGGNYAEQFF	2.10	0.00
PBS-3	CASSLRVAEVFF	34.33	0.00
	CASSLEGTAETLYF	25.68	0.00
	CASSIRGYEQYF	13.78	0.00
	CASSLVGGASYEQYF	12.02	0.00
	CGAGTGETEQFF	4.57	0.00
PBS-4	CTCSADGAGGGNERLFF	6.34	0.00
	CASSFSFSAETLYF	5.89	0.00
	CASSQTGEHTQYF	4.22	0.00
	CASSLSGSDYTF	3.73	0.00
	CASSFRGGQNTLYF	3.01	0.00
PBS-5	CASSLELGGPEQYF	7.74	0.00
	CASSDPGGSAETLYF	3.77	0.00
	CASSLGTKDTQYF	3.43	0.00
	CASRDRGTEVFF	3.03	0.00
	CASGDALGVYEQYF	2.63	0.00
Cont-VV-1	CASSLRDKKDTQYF	5.78	0.01
	CASSFTGTNNQAPLF	5.69	0.08
	CASSLRSQNTLYF	5.61	0.00
	CASSGRDRVSAEQFF	3.08	0.00
	CASSNTGYNNQAPLF	2.47	0.03
Cont-VV-2	CASSFTGRNNQAPLF	18.66	0.44
	CASSPRDRDGNTLYF	6.72	0.02
	CASSLPGQNTEVFF	3.55	0.00
	CASSLGTGSYEQYF	2.82	0.00
	CASSDAGQGAEVFF	2.28	0.00
Cont-VV-3	CASSLHSAETLYF	5.87	0.57
	CASSSTGYNNQAPLF	5.86	0.59
	CASSPGTGRNNQAPLF	3.40	0.15
	CASSRTGDSYEQYF	2.26	0.00
	CASSLVPAETLYF	1.90	0.07
Cont-VV-4	CASRGQISNERLFF	6.82	0.00
	CASSLGLGAYEQYF	3.78	0.00
	CASSIRGGRGAETLYF	3.31	0.00
	CASSSTGHNNQAPLF	3.18	0.01
	CTCSVDRVDTGQLYF	3.02	0.00
Cont-VV-5	CASSSTGENNQAPLF	7.24	0.02
	CAWSQQGRNNQAPLF	6.89	0.00
	CASGGWGGQNTLYF	6.08	0.00
	CASSLLDWGSYAEQFF	6.04	0.02
	CASSKTGGDTQYF	5.54	0.00
hIL-7-VV-1	CASSLGTTNERLFF	7.92	0.00
	CASSLGHQNTLYF	6.00	0.00
	CASSPNWGGQDTQYF	4.57	0.20
	CASSHQDTEVFF	4.18	0.00
	CASSLFDRAYAEQFF	2.98	0.03
hIL-7-VV-2	CASSQPGAYEQYF	4.78	0.00
	CASGRDFYEQYF	3.50	0.00
	CASSPGTYEQYF	2.89	0.00
	CASSRQGENNSPLYF	2.75	0.00
	CASSSTGYNNQAPLF	2.47	0.02
hIL-7-VV-3	CASSLRDWGAYAEQFF	4.85	0.00
	CASSPGLGEGEQFF	4.63	0.00
	CTCSADRQEDTQYF	2.84	0.00
	CASSPRQIQDTQYF	2.55	0.01
	CASSAGTAYEQYF	2.49	0.00
hIL-7-VV-4	CASSRQGENNQAPLF	4.24	0.03
	CASSLSYEQYF	3.58	0.00
	CASGGPYEQYF	3.58	0.00
	CTCSAYRASQNTLYF	3.47	0.00
	CASSLAWGGRRNTLYF	2.82	0.00
mIL-12-VV-1	CASSPDWGGAETLYF	9.65	0.06
	CASSSGWGRNYAEQFF	9.25	0.26
	CASSYRGLEQYF	4.42	0.26
	CASGEGYGGAQRNTLYF	3.69	2.21
	CASSLRQNSDYTF	3.57	0.15
mIL-12-VV-2	CASSIGDQDTQYF	55.79	0.00
	CASSQGNYAEQFF	28.25	0.85
	CGARVRGNSDYTF	10.98	0.44
	CASSQGNYAGQFF	0.15	0.00
	CANSIGDQDTQYF	0.14	0.00
mIL-12-VV-3	CASRTANTEVFF	36.27	3.29
	CASSLTTANTEVFF	5.04	0.06
	CASSYRDSDYTF	2.85	0.18
	CASTWGGNTLYF	2.29	0.17
	CASSPQGAETLYF	2.27	0.00
mIL-12-VV-4	CASSPDWGGAETLYF	6.75	0.13
	CASSYGGASYEQYF	5.39	0.08
	CGARQNTEVFF	4.76	1.00
	CASSLGQTANERLFF	1.75	0.08
	CASSFLGGLEQYF	1.52	0.49
hIL-7/mIL-12-VV-1	CASSLTGGGQNTLYF	12.13	0.08
	CASSQGQGSQNTLYF	11.05	0.79
	CASSTGGGYAEQFF	8.14	0.08
	CASSLELGGREQYF	6.27	0.03
	CASGDARLVSSYEQYF	4.33	0.00
hIL-7/mIL-12-VV-2	CASSVRDREDEQYF	39.77	0.00
	CASSFSPSNERLFF	25.90	0.04
	CASSGTISNERLFF	24.95	0.04
	CASSTPGTGGYEQYF	1.27	0.00
	CASSFSPANERLFF	0.23	0.00
hIL-7/mIL-12-VV-3	CASSLGTGGEEQYF	42.39	0.71
	CTCSEGWGEQNTLYF	26.86	0.00
	CASSLGVSQNTLYF	24.04	0.00
	RASSLGTGGEEQYF	0.15	0.00
	CASSVGTGGEEQYF	0.15	0.00
hIL-7/mIL-12-VV-4	CASSRQGAERLFF	26.21	0.00
	CASSSGLGEDTGQLYF	21.34	0.00
	CASSSRDRGGETLYF	8.66	0.00
	CASSRDLVSSYEQYF	5.21	0.00
	CGAKLGVQDTQYF	4.42	0.14
hIL-7/mIL-12-VV-5	CASSPGTSSQNTLYF	10.14	0.03
	CASSQTRDWGYEQYF	5.51	0.57
	CASSPNWGEGDTQYF	3.87	0.06
	CASSPPGGDEQYF	3.65	0.19
	CASSLLNYAEQFF	2.51	0.21

Information about TRBV, TRBJ, the number of individuals reads, and the number of total in-frame reads in each sample are described in Supplementary Data File 1

## Discussion

Immune checkpoint inhibitors have markedly changed cancer treatment in the past decade, and their use in preclinical and clinical studies has dramatically improved our understanding of complicated immune mechanisms in the tumor microenvironment. However, because patient response rates remain low, various types of immunotherapies are currently being assessed to further improve efficacy while minimizing immune-related adverse events. Among these, oncolytic virotherapy is a promising monotherapy and combinatorial therapy with immune checkpoint inhibitors due to its ability to transform “cold” tumors to “hot” tumors by increasing activated T cells, natural killer (NK) cells and inflammatory cytokines, which augment the efficacy of immune checkpoint blockade [28]. Moreover, studies on oncolytic viruses carrying immunomodulator(s) are being actively conducted with the aim to further activate the immune system to overcome the immunosuppressive tumor microenvironment [29]. We recently reported that simultaneous expression of IL-7 and IL-12 in poorly immunogenic LLC tumors showed superior antitumor activity to expression of either cytokine alone [15]. In this study, we demonstrated that expression of IL-7 together with IL-12 increased a limited number of T cell clones in tumors, which is one crucial mechanism underlying the difference in antitumor efficacy between IL-12 alone and the combination of IL-7 and IL-12. Further, our data demonstrated that IL-7 has opposing effects on the TCR repertoire of intratumoral CD8^+^ T cells in the presence compared to the absence of IL-12.

IL-12 is a well-studied proinflammatory cytokine that efficiently promotes antitumor immune responses due to its ability to establish a link between innate and adaptive immunity by activating NK cells and T cells [30]. Effects of exogenous IL-12 expression are currently being studied in mice, non-human primates and humans [29, 31, 32]. Moreover, IL-12-based combination therapies with other anticancer agents are under investigation [33, 34]. Few studies have investigated the combination of IL-12 and IL-7, which is essential for maintaining naïve CD4^+^ and CD8^+^ T cells and the survival of antigen-specific memory T cells [35–37]. IL-7 has been reported to synergistically stimulate T cells in vitro when combined with IL-12 [38]. Our data showed that intratumoral dual expression of IL-7 and IL-12, compared to IL-12 alone, increased activated CD8^+^ T cells in poorly immunogenic tumors, which is supported by findings from previous reports. It is assumed that synergistically stimulated T cells in tumors in the presence of IL-7 and IL-12 may further upregulate multiple immune pathways, enhancing inflammatory status, and leading to antitumor efficacy. Moreover, we found that IL-7 combined with IL-12 did not increase the proportion of exhausted CD8^+^ T cells, despite T cell activation, in which IL-7 is assumed to contribute to maintaining T cells in the tumor microenvironment.

However, these findings focusing on T cell activation are not sufficient to explain the difference in the proportion of mice that achieved CR following treatment with mIL-12-VV compared to hIL-7/mIL-12-VV. Differences in efficacy always emerged about two weeks after viral treatment, when little to no virus was present in tumors and the concentrations of IL-7, IL-12 and IFNγ were below the level of quantification (unpublished data). IL-7 is known to be required for the development of DCs [39] and, furthermore, for efficient interaction between T cells and DCs [40]. Our NanoString gene analysis of hIL-7/mIL-12-VV-treated tumors and mIL-12-VV-treated tumors demonstrated a difference in expression levels of APC-related genes, which led us to hypothesize that IL-7 contributes to induction of more efficient adaptive immunity. Thus, we investigated factors that have potential long-term effects, such as TCR clonality in tumors and the spleen. Intratumoral administration of Cont-VV increased the diversity of CD8^+^ and CD4^+^ T cells, which is in agreement with prior reports showing that vaccine therapy and in situ vaccination with radiotherapy increases the number of T cell clones [13, 14]. Intratumoral administration of hIL-7-VV also increased diversity, a result that is consistent with reports showing that administration of recombinant human IL-7 to non-human primates or humans resulted in an increase in T cell number and increased TCR diversity [41, 42]. However, when administered with IL-12, IL-7 markedly increased the clonality of CD8^+^ T cells, resulting in a higher proportion of mice treated with hIL-7/mIL-12-VV than mIL-12-VV showing multiple high-frequency clones in tumors, which seemed to correlate with the proportion of mice that achieved complete tumor regression. It is plausible that CD8^+^ T cells in tumors treated with hIL-7/mIL-12-VV are highly regulated by IL-7, which can promote proliferation of antigen-specific memory T cells in the presence of tumor antigens, preventing apoptosis of T cells, while IL-7 alone without tumor antigens has lower potential for memory T cell proliferation [37].

We found some T cell clones in spleens which had been identified as high-frequency clones in tumors, suggesting migration of T cells from virus-injected tumors to secondary lymphoid organs. However, T cell diversity in the spleen did not clearly reflect that in tumors. This indicates that local changes in immune status are likely to be sufficient for antitumor efficacy, and our recombinant oncolytic vaccinia viruses successfully played their role with minimum safety concerns due to the tumor selectivity of viral replication [15]. However, these findings also suggest the difficulty of using peripheral TCR repertoire analysis as a precise response biomarker during therapy. Even in patients treated with immune checkpoint inhibitors, there are currently many uncertainties regarding the relationship between the peripheral T cell repertoire and antitumor efficacy [11, 43–45]. Identification of a parameter which reflects the status of the intratumoral TCR repertoire is important for predicting patient outcome.

We acknowledge that there are several limitations to this study that prevent full profiling of the mechanism underlying this virotherapy. First, related to the difficulty of using TCR repertoire analysis for diagnosis mentioned above, it is not possible to clearly prove that individual mice with multiple high-frequency clones in tumors after treatment with hIL-7/mIL-12-VV indeed have the potential to achieve CR. Second, it is unclear whether the TCR of each high-frequency clone in tumors recognizes tumor antigens, viral proteins or other molecules, despite our tumor rechallenge study suggesting the existence of T cell clones that are reactive to tumor antigens. Third, while we described a bipolar function for IL-7 on CD8^+^ T cells in the presence of IL-12, it will be important to investigate why this is not observed on CD4^+^ T cells by conducting detailed analysis of the mechanisms, with a focus on the differences between CD8^+^ and CD4^+^ T cells.

In summary, we demonstrated that intratumoral expression of IL-7 together with IL-12 increases the intratumoral clonality of CD8^+^ T cells and improves the antitumor response rate in a poorly immunogenic mouse model, thereby revealing an unknown function of IL-7 that is triggered by IL-12. Our data provide a scientific rationale for evaluating IL-7 and IL-12 combination virotherapy in humans, and may further improve our understanding of cancer immunology.

### Supplementary information

Below is the link to the electronic supplementary material.

Supplementary file1 (PDF 62 kb)

Supplementary file2 (PDF 62 kb)

Supplementary file3 (PDF 288 kb)

## Data Availability

All data associated with this study are present in the paper or the Supplementary Materials. Correspondence and requests for materials should be addressed to Shinsuke Nakao.
