# Supplementary material for: IL-7 coupled with IL-12 increases intratumoral T cell clonality, leading to complete regression of non-immunogenic tumors
Source: Cancer Immunol Immunother. 2021 Apr 28;70(12):3557–71. doi: 10.1007/s00262-021-02947-y (PMC8571137; doi:10.1007/s00262-021-02947-y)
Supplement: Supplementary file 1 — Supplementary file1 (PDF 62 kb) [file 262_2021_2947_MOESM1_ESM.pdf]

Mouse	Top 5 CD8+ T-cell clones in tumors				Spleen specimens		
	TRBV	TRBJ	CDR3β resion sequence	frequency (%)	Total in-frame reads	reads	Frequency (%)
PBS-1	TRBV20	TRBJ1-1	CGARQSNTEVFF	3.93	149479	0	0.000
	TRBV16	TRBJ2-7	CASSLDWGDYEQYF	3.02		0	0.000
	TRBV29	TRBJ2-7	CASRQGEQYF	2.91		0	0.000
	TRBV5	TRBJ2-5	CASSQVLGDTQYF	2.76		0	0.000
	TRBV12-2	TRBJ2-1	CASSPDWVYAEQFF	2.50		0	0.000
PBS-2	TRBV16	TRBJ2-5	CASSLELGGPTQYF	14.41	214985	0	0.000
	TRBV5	TRBJ2-1	CASSQEQTINYAEQFF	3.15		0	0.000
	TRBV19	TRBJ1-1	CASSIKVANTEVFF	2.29		0	0.000
	TRBV15	TRBJ2-7	CASSLLGAYEQYF	2.26		44	0.020
	TRBV12-2	TRBJ2-1	CASSPTGGNYAEQFF	2.10		0	0.000
PBS-3	TRBV12-1	TRBJ1-1	CASSLRVAEVFF	34.33	98053	0	0.000
	TRBV16	TRBJ2-3	CASSLEGTAETLYF	25.68		0	0.000
	TRBV19	TRBJ2-7	CASSIRGYEQYF	13.78		0	0.000
	TRBV15	TRBJ2-7	CASSLVGGASYEQYF	12.02		0	0.000
	TRBV20	TRBJ2-1	CGAGTGETEQFF	4.57		0	0.000
PBS-4	TRBV1	TRBJ1-4	CTCSADGAGGGNERLFF	6.34	174695	0	0.000
	TRBV3	TRBJ2-3	CASSFSFSAETLYF	5.89		0	0.000
	TRBV12-2	TRBJ2-5	CASSQTGEHTQYF	4.22		0	0.000
	TRBV12-2	TRBJ1-2	CASSLSGSDYTF	3.73		0	0.000
	TRBV14	TRBJ2-4	CASSFRGGQNTLYF	3.01		0	0.000
PBS-5	TRBV16	TRBJ2-7	CASSLELGGPEQYF	7.74	188571	0	0.000
	TRBV13-1	TRBJ2-3	CASSDPGGSAETLYF	3.77		0	0.000
	TRBV12-2	TRBJ2-5	CASSLGTKDTQYF	3.43		0	0.000
	TRBV26	TRBJ1-1	CASRDRGTEVFF	3.03		0	0.000
	TRBV13-2	TRBJ2-7	CASGDALGVYEQYF	2.63		0	0.000
Cont-VV-1	TRBV15	TRBJ2-5	CASSLRDKKDTQYF	5.78	123343	9	0.007
	TRBV14	TRBJ1-5	CASSFTGTNNQAPLF	5.69		95	0.077
	TRBV26	TRBJ2-4	CASSLRSQNTLYF	5.61		0	0.000
	TRBV17	TRBJ2-1	CASSGRDRVSAEQFF	3.08		0	0.000
	TRBV14	TRBJ1-5	CASSNTGYNNQAPLF	2.47		42	0.034
Cont-VV-2	TRBV14	TRBJ1-5	CASSFTGRNNQAPLF	18.66	134999	600	0.444
	TRBV14	TRBJ1-3	CASSPRDRDGNTLYF	6.72		26	0.019
	TRBV15	TRBJ1-1	CASSLPGQNTEVFF	3.55		0	0.000
	TRBV3	TRBJ2-7	CASSLGTGSYEQYF	2.82		0	0.000
	TRBV13-1	TRBJ1-1	CASSDAGQGAEVFF	2.28		0	0.000
Cont-VV-3	TRBV14	TRBJ2-3	CASSLHSAETLYF	5.87	136749	775	0.567
	TRBV14	TRBJ1-5	CASSSTGYNNQAPLF	5.86		806	0.589
	TRBV3	TRBJ1-5	CASSPGTGRNNQAPLF	3.40		210	0.154
	TRBV12-1	TRBJ2-7	CASSRTGDSYEQYF	2.26		0	0.000
	TRBV12-2	TRBJ2-3	CASSLVPAETLYF	1.90		102	0.075
Cont-VV-4	TRBV13-3	TRBJ1-4	CASRGQISNERLFF	6.82	94574	0	0.000
	TRBV12-1	TRBJ2-7	CASSLGLGAYEQYF	3.78		0	0.000
	TRBV19	TRBJ2-3	CASSIRGGRGAETLYF	3.31		0	0.000
	TRBV14	TRBJ1-5	CASSSTGHNNQAPLF	3.18		9	0.010
	TRBV1	TRBJ2-2	CTCSVDRVDTGQLYF	3.02		0	0.000
Cont-VV-5	TRBV14	TRBJ1-5	CASSSTGENNQAPLF	7.24	166299	26	0.016
	TRBV31	TRBJ1-5	CAWSQQGRNNQAPLF	6.89		0	0.000
	TRBV13-2	TRBJ2-4	CASGGWGGQNTLYF	6.08		0	0.000
	TRBV12-2	TRBJ2-1	CASSLLDWGSYAEQFF	6.04		37	0.022
	TRBV17	TRBJ2-5	CASSKTGGDTQYF	5.54		0	0.000
hIL-7-VV-1	TRBV15	TRBJ1-4	CASSLGTTNERLFF	7.92	154527	0	0.000
	TRBV15	TRBJ2-4	CASSLGHQNTLYF	6.00		0	0.000
	TRBV5	TRBJ2-5	CASSPNWGGQDTQYF	4.57		304	0.197
	TRBV2	TRBJ1-1	CASSHQDTEVFF	4.18		0	0.000
	TRBV29	TRBJ2-1	CASSLFDRAYAEQFF	2.98		48	0.031
hIL-7-VV-2	TRBV5	TRBJ2-7	CASSQPGAYEQYF	4.78	244335	0	0.000
	TRBV13-2	TRBJ2-7	CASGRDFYEQYF	3.50		0	0.000
	TRBV4	TRBJ2-7	CASSPGTYEQYF	2.89		0	0.000
	TRBV4	TRBJ1-6	CASSRQGENNSPLYF	2.75		0	0.000
	TRBV14	TRBJ1-5	CASSSTGYNNQAPLF	2.47		37	0.015
hIL-7-VV-3	TRBV26	TRBJ2-1	CASSLRDWGAYAEQFF	4.85	182909	0	0.000
	TRBV16	TRBJ2-1	CASSPGLGEGEQFF	4.63		0	0.000
	TRBV1	TRBJ2-5	CTCSADRQEDTQYF	2.84		0	0.000
	TRBV29	TRBJ2-5	CASSPRQIQDTQYF	2.55		21	0.011
	TRBV17	TRBJ2-7	CASSAGTAYEQYF	2.49		0	0.000
hIL-7-VV-4	TRBV4	TRBJ1-5	CASSRQGENNQAPLF	4.24	211739	66	0.031
	TRBV4	TRBJ2-7	CASSLSYEQYF	3.58		0	0.000
	TRBV13-2	TRBJ2-7	CASGGPYEQYF	3.58		0	0.000
	TRBV1	TRBJ2-4	CTCSAYRASQNTLYF	3.47		0	0.000
	TRBV12-2	TRBJ2-4	CASSLAWGGRRNTLYF	2.82		0	0.000

Mouse	Top 5 CD8+ T-cell clones in tumors				Spleen specimens		
	TRBV	TRBJ	CDR3β resion sequence	frequency (%)	Total in-frame reads	reads	Frequency (%)
mIL-12-VV-1	TRBV3	TRBJ2-3	CASSPDWGGAETLYF	9.65	179484	100	0.056
	TRBV12-2	TRBJ2-1	CASSSGWGRNYAEQFF	9.25		470	0.262
	TRBV13-3	TRBJ2-7	CASSYRGLEQYF	4.42		462	0.257
	TRBV13-2	TRBJ2-4	CASGEGYGGAQRNTLYF	3.69		3970	2.212
	TRBV26	TRBJ1-2	CASSLRQNSDYTF	3.57		270	0.150
mIL-12-VV-2	TRBV19	TRBJ2-5	CASSIGDQDTQYF	55.79	132120	0	0.000
	TRBV13-1	TRBJ2-1	CASSQGNYAEQFF	28.25		1118	0.846
	TRBV20	TRBJ1-2	CGARVRGNSDYTF	10.98		580	0.439
	TRBV13-1	TRBJ2-1	CASSQGNYAGQFF	0.15		1	0.001
	TRBV19	TRBJ2-5	CANSIGDQDTQYF	0.14		0	0.000
mIL-12-VV-3	TRBV13-3	TRBJ1-1	CASRTANTEVFF	36.27	126558	4158	3.285
	TRBV3	TRBJ1-1	CASSLTTANTEVFF	5.04		73	0.058
	TRBV13-3	TRBJ1-2	CASSYRDSDYTF	2.85		222	0.175
	TRBV19	TRBJ2-4	CASTWGGNTLYF	2.29		217	0.171
	TRBV26	TRBJ2-3	CASSPQGAETLYF	2.27		0	0.000
mIL-12-VV-4	TRBV3	TRBJ2-3	CASSPDWGGAETLYF	6.75	185935	243	0.131
	TRBV4	TRBJ2-7	CASSYGGASYEQYF	5.39		154	0.083
	TRBV20	TRBJ1-1	CGARQNTEVFF	4.76		1867	1.004
	TRBV3	TRBJ1-4	CASSLGQTANERLFF	1.75		140	0.075
	TRBV14	TRBJ2-7	CASSFLGGLEQYF	1.52		907	0.488
hIL-7/mIL-12-VV-1	TRBV13-1	TRBJ2-4	CASSLTGGGQNTLYF	12.13	160466	126	0.079
	TRBV5	TRBJ2-4	CASSQGQGSQNTLYF	11.05		1266	0.789
	TRBV13-1	TRBJ2-1	CASSTGGGYAEQFF	8.14		132	0.082
	TRBV16	TRBJ2-7	CASSLELGGREQYF	6.27		49	0.031
	TRBV13-2	TRBJ2-7	CASGDARLVSSYEQYF	4.33		0	0.000
hIL-7/mIL-12-VV-2	TRBV13-1	TRBJ2-7	CASSVRDREDEQYF	39.77	131217	0	0.000
	TRBV29	TRBJ1-4	CASSFSPSNERLFF	25.90		57	0.043
	TRBV13-3	TRBJ1-4	CASSGTISNERLFF	24.95		57	0.043
	TRBV4	TRBJ2-7	CASSTPGTGGYEQYF	1.27		0	0.000
	TRBV29	TRBJ1-4	CASSFSPANERLFF	0.23		0	0.000
hIL-7/mIL-12-VV-3	TRBV14	TRBJ2-7	CASSLGTGGEEQYF	42.39	352049	2484	0.706
	TRBV1	TRBJ2-4	CTCSEGWGEQNTLYF	26.86		0	0.000
	TRBV14	TRBJ2-4	CASSLGVSQNTLYF	24.04		0	0.000
	TRBV14	TRBJ2-7	RASSLGTGGEEQYF	0.15		1	0.000
	TRBV14	TRBJ2-7	CASSVGTGGEEQYF	0.15		1	0.000
hIL-7/mIL-12-VV-4	TRBV5	TRBJ1-4	CASSRQGAERLFF	26.21	154362	0	0.000
	TRBV14	TRBJ2-2	CASSSGLGEDTGQLYF	21.34		0	0.000
	TRBV16	TRBJ2-3	CASSSRDRGGETLYF	8.66		0	0.000
	TRBV17	TRBJ2-7	CASSRDLVSSYEQYF	5.21		0	0.000
	TRBV20	TRBJ2-5	CGAKLGVQDTQYF	4.42		216	0.140
hIL-7/mIL-12-VV-5	TRBV14	TRBJ2-4	CASSPGTSSQNTLYF	10.14	205548	65	0.032
	TRBV5	TRBJ2-7	CASSQTRDWGYEQYF	5.51		1177	0.573
	TRBV13-3	TRBJ2-5	CASSPNWGEGDTQYF	3.87		125	0.061
	TRBV5	TRBJ2-7	CASSPPGGDEQYF	3.65		395	0.192
	TRBV29	TRBJ2-1	CASSLLNYAEQFF	2.51		440	0.214
