# Supplementary material for: IL-7 coupled with IL-12 increases intratumoral T cell clonality, leading to complete regression of non-immunogenic tumors
Source: Cancer Immunol Immunother. 2021 Apr 28;70(12):3557–71. doi: 10.1007/s00262-021-02947-y (PMC8571137; doi:10.1007/s00262-021-02947-y)
Supplement: Supplementary file 2 — Supplementary file2 (PDF 62 kb) [file 262_2021_2947_MOESM2_ESM.pdf]

Mouse	Top 5 CD4+ T-cell clones in tumors				Spleen specimens		
	TRBV	TRBJ	CDR3β resion sequence	frequency (%)	Total in-frame reads	reads	Frequency (%)
PBS-1	TRBV19	TRBJ2-1	CASSPLGYAEQFF	3.16	194738	0	0.000
	TRBV26	TRBJ1-4	CASSLSRGPNERLFF	3.13		0	0.000
	TRBV26	TRBJ1-3	CASSLAGSGNTLYF	2.83		0	0.000
	TRBV5	TRBJ1-1	CASSQPGQTEVFF	2.56		0	0.000
	TRBV29	TRBJ2-7	CASSLPGGYEQYF	2.42		0	0.000
PBS-2	TRBV12-2	TRBJ2-7	CASSHRGEQYF	4.08	116960	0	0.000
	TRBV26	TRBJ1-6	CASSLSRGGSPLYF	2.63		2	0.002
	TRBV26	TRBJ2-1	CASSRTDNYAEQFF	2.61		1	0.001
	TRBV1	TRBJ2-7	CTCSAPDNYEQYF	2.61		13	0.011
	TRBV16	TRBJ2-5	CASSFFNQDTQYF	2.25		1	0.001
PBS-3	TRBV13-1	TRBJ2-7	CASSDEGRAYEQYF	9.67	203738	0	0.000
	TRBV5	TRBJ1-3	CASSQENSGNTLYF	8.44		0	0.000
	TRBV16	TRBJ2-4	CASSLDDQNTLYF	7.65		0	0.000
	TRBV26	TRBJ2-4	CASRDRGGGQNTLYF	7.34		0	0.000
	TRBV26	TRBJ2-1	CASSPDRDAEQFF	5.69		69	0.034
PBS-4	TRBV12-2	TRBJ2-4	CASSPPGGWNTLYF	7.82	173960	0	0.000
	TRBV1	TRBJ1-2	CTCSATGTGNSDYTF	6.60		0	0.000
	TRBV3	TRBJ1-3	CASSPQGPGNTLYF	3.58		0	0.000
	TRBV13-2	TRBJ2-7	CASGVGGRDEQYF	2.19		0	0.000
	TRBV13-3	TRBJ1-4	CASSLGQLSNERLFF	1.96		0	0.000
PBS-5	TRBV13-2	TRBJ2-1	CASGEGLGGLAEQFF	8.22	197775	422	0.213
	TRBV5	TRBJ2-4	CASSLDISQNTLYF	2.34		0	0.000
	TRBV16	TRBJ2-7	CASSLELGGPEQYF	2.20		0	0.000
	TRBV23	TRBJ2-7	CSSSPSGGAYEQYF	2.13		1152	0.582
	TRBV5	TRBJ2-7	CASSQDPGGFYEQYF	1.55		0	0.000
Cont-VV-1	TRBV1	TRBJ1-3	CTCSAGGSGNTLYF	9.43	162775	0	0.000
	TRBV15	TRBJ1-1	CASSPTGNTEVFF	1.25		0	0.000
	TRBV15	TRBJ1-6	CASSQLNF	1.23		0	0.000
	TRBV15	TRBJ2-2	CASSLWGGAGTGQLYF	1.09		35	0.022
	TRBV2	TRBJ1-6	CASSQDPYNSPLYF	1.04		0	0.000
Cont-VV-2	TRBV5	TRBJ2-7	CASSQEGLGGREQYF	1.93	214434	0	0.000
	TRBV19	TRBJ1-3	CASSISRSGNTLYF	1.77		339	0.158
	TRBV14	TRBJ1-2	CASSFQGSNSDYTF	1.65		23	0.011
	TRBV1	TRBJ2-4	CTCSAGRGSQNTLYF	1.55		71	0.033
	TRBV12-1	TRBJ2-1	CASSLRDWGVAEQFF	1.50		0	0.000
Cont-VV-3	TRBV29	TRBJ2-5	CASSLQGGRDTQYF	1.63	83819	127	0.152
	TRBV15	TRBJ1-1	CASSLQGGTEVFF	1.59		0	0.000
	TRBV16	TRBJ2-4	CASSLEGDTLYF	1.50		87	0.104
	TRBV5	TRBJ1-1	CASSQVNTEVFF	1.33		0	0.000
	TRBV17	TRBJ1-1	CASSRRDRATEVFF	1.33		27	0.032
Cont-VV-4	TRBV12-2	TRBJ2-7	CASSLTQEQYF	1.74	215054	69	0.032
	TRBV13-2	TRBJ2-5	CASGLDWGGDQDTQYF	1.06		0	0.000
	TRBV26	TRBJ2-4	CASSLDWGSQNTLYF	0.95		0	0.000
	TRBV5	TRBJ1-1	CASSPGQNTEVFF	0.90		0	0.000
	TRBV29	TRBJ2-7	CASSPGTGGYEQYF	0.90		0	0.000
Cont-VV-5	TRBV12-2	TRBJ2-4	CASSLESQNTLYF	2.72	72737	0	0.000
	TRBV12-2	TRBJ1-3	CASSLSGNTLYF	2.26		0	0.000
	TRBV23	TRBJ1-1	CSSSQLWANTEVFF	1.43		3	0.004
	TRBV19	TRBJ2-7	CASSIGTGAYEQYF	1.27		0	0.000
	TRBV3	TRBJ2-5	CASSLDWGQDTQYF	1.18		0	0.000
hIL-7-VV-1	TRBV16	TRBJ1-1	CASSLGQNTEVFF	1.81	254597	13	0.005
	TRBV13-2	TRBJ1-1	CASGDEAGQTNTEVFF	1.60		0	0.000
	TRBV12-2	TRBJ1-5	CASSRQDQAPLF	1.43		0	0.000
	TRBV19	TRBJ2-5	CASSRMEDTQYF	1.24		35	0.014
	TRBV12-2	TRBJ2-5	CASSPTGDQDTQYF	1.00		0	0.000
hIL-7-VV-2	TRBV5	TRBJ2-2	CASSPGTVNTGQLYF	1.37	247258	0	0.000
	TRBV26	TRBJ2-4	CASSRDWGSQNTLYF	1.19		23	0.009
	TRBV1	TRBJ2-3	CTCSAVGGGAETLYF	1.07		466	0.188
	TRBV26	TRBJ1-1	CASSPGPNTEVFF	1.03		0	0.000
	TRBV12-2	TRBJ1-4	CASSRSNERLFF	0.80		0	0.000
hIL-7-VV-3	TRBV26	TRBJ1-2	CASSLGTGGSDYTF	1.79	202748	5	0.002
	TRBV13-2	TRBJ1-1	CASGGGANTEVFF	1.68		0	0.000
	TRBV16	TRBJ1-1	CASSLGQNTEVFF	1.30		0	0.000
	TRBV13-3	TRBJ1-4	CASSHRAERLFF	1.30		0	0.000
	TRBV5	TRBJ2-2	CASSQEGINTGQLYF	1.19		0	0.000
hIL-7-VV-4	TRBV13-2	TRBJ1-1	CASGDAYITEVFF	2.19	256586	9	0.004
	TRBV3	TRBJ2-7	CASSLALWGYEQYF	1.11		0	0.000
	TRBV5	TRBJ1-1	CASSQQGWEVFF	1.11		0	0.000
	TRBV5	TRBJ1-4	CASSQEGTGNERLFF	1.07		164	0.064
	TRBV23	TRBJ2-3	CSSSRQGSETLYF	1.04		0	0.000

Mouse	Top 5 CD4+ T-cell clones in tumors				Spleen specimens		
	TRBV	TRBJ	CDR3β resion sequence	frequency (%)	Total in-frame reads	reads	Frequency (%)
mIL-12-VV-1	TRBV1	TRBJ2-7	CTCSAGQGWEQYF	8.51	186216	0	0.000
	TRBV16	TRBJ2-5	CASSLEGGQDTQYF	7.60		329	0.177
	TRBV1	TRBJ2-5	CTCSALGTSQDTQYF	6.25		0	0.000
	TRBV13-1	TRBJ1-1	CASSDGGTEVFF	5.31		0	0.000
	TRBV17	TRBJ2-7	CASSPDWASSYEQYF	4.98		0	0.000
mIL-12-VV-2	TRBV13-2	TRBJ1-2	CASGDADRRGSDYTF	89.65	125750	0	0.000
	TRBV13-2	TRBJ1-2	WASGDADRRGSDYTF	0.48		0	0.000
	TRBV13-2	TRBJ1-2	CASGDADRRGSDYTV	0.31		0	0.000
	TRBV13-2	TRBJ1-2	CAGGDADRRGSDYTF	0.25		0	0.000
	TRBV13-2	TRBJ1-2	CASGDVDRRGSDYTF	0.20		0	0.000
mIL-12-VV-3	TRBV13-3	TRBJ1-1	CASSGQGNTEVFF	6.45	184538	547	0.296
	TRBV3	TRBJ2-5	CASSRDWGDTQYF	6.30		890	0.482
	TRBV15	TRBJ2-7	CASSPMTGGEQYF	6.30		0	0.000
	TRBV3	TRBJ2-5	CASSLETDNQDTQYF	5.80		0	0.000
	TRBV17	TRBJ2-4	CASSRLGVQNTLYF	5.29		180	0.098
mIL-12-VV-4	TRBV16	TRBJ2-4	CASSLEGQNTLYF	16.07	153242	113	0.074
	TRBV13-2	TRBJ2-7	CASGDAWGGYEQYF	7.79		1	0.001
	TRBV20	TRBJ2-7	CGARETGDEQYF	5.39		56	0.037
	TRBV15	TRBJ1-4	CASRTAERLFF	5.14		0	0.000
	TRBV16	TRBJ1-4	CASSLEGGSNERLFF	4.78		43	0.028
hIL-7/mIL-12-VV-1	TRBV4	TRBJ2-5	CASRVRGRDTQYF	6.53	232697	7	0.003
	TRBV14	TRBJ2-1	CASSQTYNYAEQFF	5.44		59	0.025
	TRBV16	TRBJ2-5	CASSLEGGEDTQYF	3.22		10	0.004
	TRBV13-2	TRBJ2-7	CASGDAGGALGEQYF	3.20		0	0.000
	TRBV1	TRBJ1-1	CTCSADPGTGTEVFF	2.17		0	0.000
hIL-7/mIL-12-VV-2	TRBV31	TRBJ1-1	CAWSLMRDTEVFF	43.62	169089	0	0.000
	TRBV12-2	TRBJ2-7	CASSLWGGAEQYF	43.07		0	0.000
	TRBV16	TRBJ2-2	CASSLDTNTGQLYF	5.45		0	0.000
	TRBV12-2	TRBJ2-7	CASSRWGGAEQYF	0.21		0	0.000
	TRBV12-2	TRBJ2-7	CASALWGGAEQYF	0.19		0	0.000
hIL-7/mIL-12-VV-3	TRBV15	TRBJ1-1	CASSFYVFF	49.60	179748	1	0.001
	TRBV13-1	TRBJ2-3	CASSDPGGTETLYF	31.61		53	0.029
	TRBV1	TRBJ2-4	CTCSATGEGNTLYF	13.00		0	0.000
	TRBV15	TRBJ1-1	CASSCYVFF	0.17		0	0.000
	TRBV15	TRBJ1-1	CASSVYVFF	0.14		0	0.000
hIL-7/mIL-12-VV-4	TRBV14	TRBJ2-4	CASSYSQNTLYF	13.90	192924	53	0.027
	TRBV13-2	TRBJ1-5	CASGGGGDNNQAPLF	10.96		0	0.000
	TRBV19	TRBJ1-6	CASSLQGFYNSPLYF	3.25		19	0.010
	TRBV31	TRBJ2-5	CAWSLGLGDQDTQYF	3.19		0	0.000
	TRBV1	TRBJ2-7	CTCSADGTGDYEQYF	3.19		27	0.014
hIL-7/mIL-12-VV-5	TRBV14	TRBJ2-4	CASSLPGQGGSQNTLYF	2.92	226968	286	0.126
	TRBV2	TRBJ1-4	CASSQPGQTNERLFF	1.77		0	0.000
	TRBV23	TRBJ2-7	CSSSQGTGGYEQYF	1.30		69	0.030
	TRBV13-2	TRBJ2-2	CASGDWGANTGQLYF	1.30		1	0.000
	TRBV31	TRBJ1-2	CAWSLGIANSDYTF	1.29		13	0.006
