# Supplementary material for: IL-7 coupled with IL-12 increases intratumoral T cell clonality, leading to complete regression of non-immunogenic tumors
Source: Cancer Immunol Immunother. 2021 Apr 28;70(12):3557–71. doi: 10.1007/s00262-021-02947-y (PMC8571137; doi:10.1007/s00262-021-02947-y)
Supplement: Supplementary file 3 — Supplementary file3 (PDF 288 kb) [file 262_2021_2947_MOESM3_ESM.pdf]

Supplementary Figure 1.

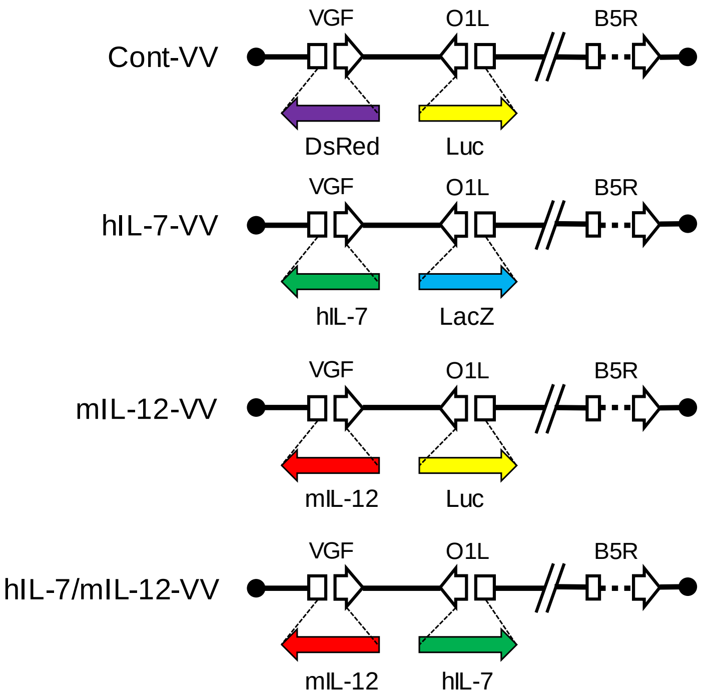

Schematic diagram of recombinant vaccinia viruses. Recombinant vaccinia viruses carrying transgenes expressing DsRed and luciferase (Cont-VV); human IL-7 and LacZ (hIL-7-VV); murine IL-12 and luciferase (mIL-12-VV); and human IL-7 and murine IL-12 (hIL-7/mIL-12-VV) were used in this study. Based on LC16mO, an attenuated vaccine strain, the viruses were modified with a functional deletion of vaccinia virus growth factor (VGF) and O1L by inserting the indicated transgenes with the aim of inducing tumor-selective viral replication. In addition, the B5R membrane protein was partially deleted with the aim of reducing antigenicity.

Supplementary Figure 2.

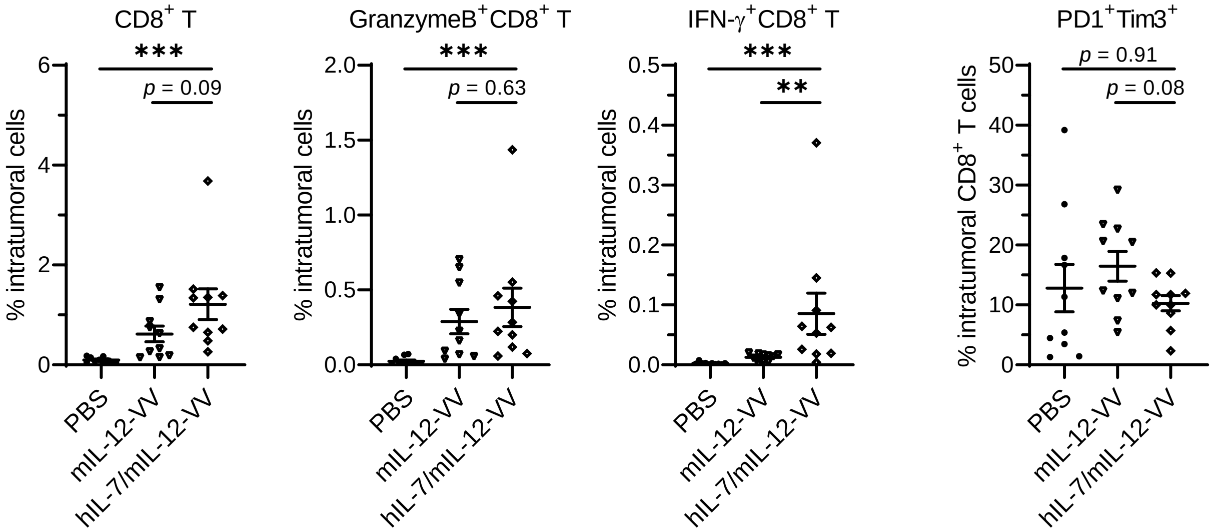

Increase in activated CD8+ T cells in LLC tumors at different time points after treatment with mIL-12-VV or hIL-7/mIL-12-VV. Mice bearing subcutaneous LLC tumors were intratumorally injected with PBS, 2×10^7 pfu of mIL-12-VV or hIL-7/mIL-12-VV for a total of three times (on Days 1, 3 and 4). Twelve days after the last treatment (on Day 16), tumor-infiltrating CD8+ T cells were analyzed by flow cytometry (n = 10). **$p < 0.01$ and ***$p < 0.001$ by Mann-Whitney *U* test. Mean ± SEM.

Supplementary Figure 3.

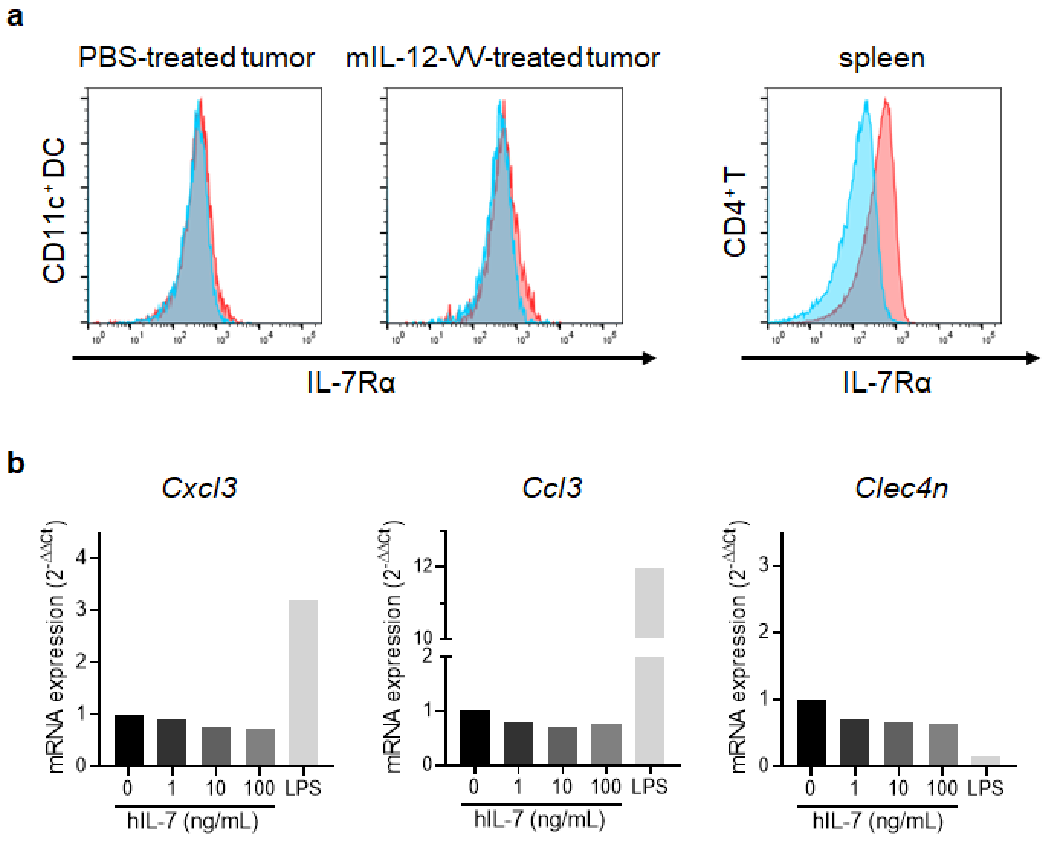

Effect of IL-7 on LLC-derived DCs. **a** Expression of IL-7Rα in intratumoral CD11c$^+$ DCs and splenic CD4$^+$ T cells. Mice bearing subcutaneous LLC tumors were intratumorally injected with PBS or 2 × 10^7 pfu of mIL-12-VV. Two days after treatment, intratumoral CD11c$^+$ DCs were analyzed by flow cytometry. Likewise, splenic CD4$^+$ T cells were analyzed as a positive control for IL-7Rα staining. Red: expression intensity of IL-7Rα, blue: control. Representative histograms are shown. **b** Effect of IL-7 on gene expression of *Cxcl3*, *Ccl3* and *Clec4n* in CD11c$^+$ DCs isolated from LLC tumors which had been treated with mIL-12-VV as described above. Gene expression levels normalized to *β-actin* are shown.

Supplementary Figure 4.

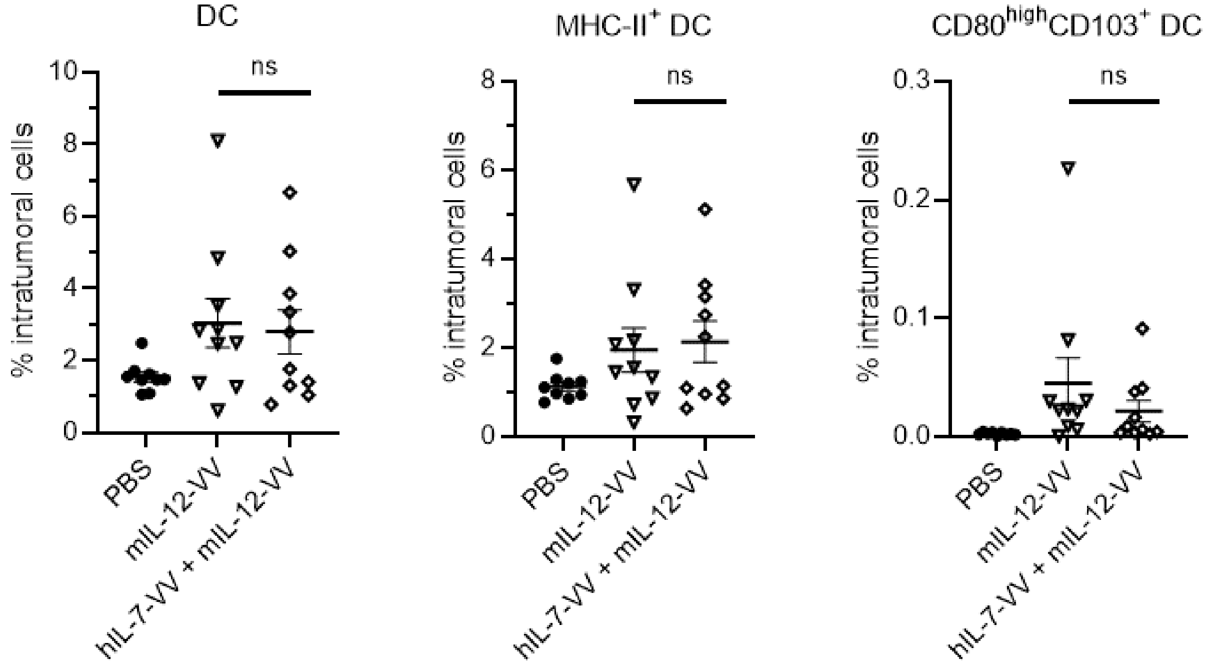

Percentage of DCs in LLC tumors treated with mIL-12-VV alone or in combination with hIL-7-VV. Mice bearing subcutaneous LLC tumors (about 70 mm^3) were intratumorally injected with PBS, mIL-12-VV (2×10^7 pfu, supplemented with 2×10^7 pfu of Cont-VV) or a combination of hIL-7-VV and mIL-12-VV (2×10^7 pfu each) every other day for a total of three times. Ten days after the last treatment, DCs, MHC class II-expressing mature DCs and $CD80^+CD103^+$ DCs were analyzed by flow cytometry (n = 9 to 10). Mean ± SEM is shown. ns: no significance between mIL-12-VV alone and in combination with hIL-7-VV by Mann-Whitney *U* test.

Supplementary Figure 5.

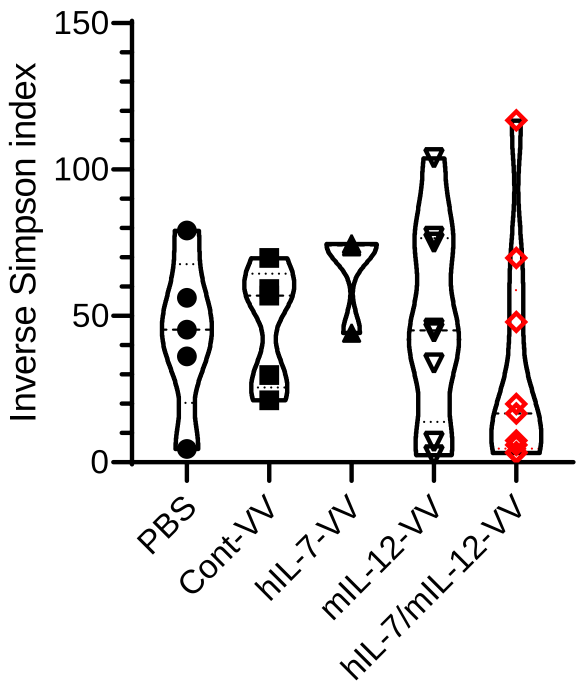

Clonality index (inverse Simpson's index) of intratumoral $CD8^+$ T cells in each treatment group, as described in Figure 2.

Supplementary Figure 6.

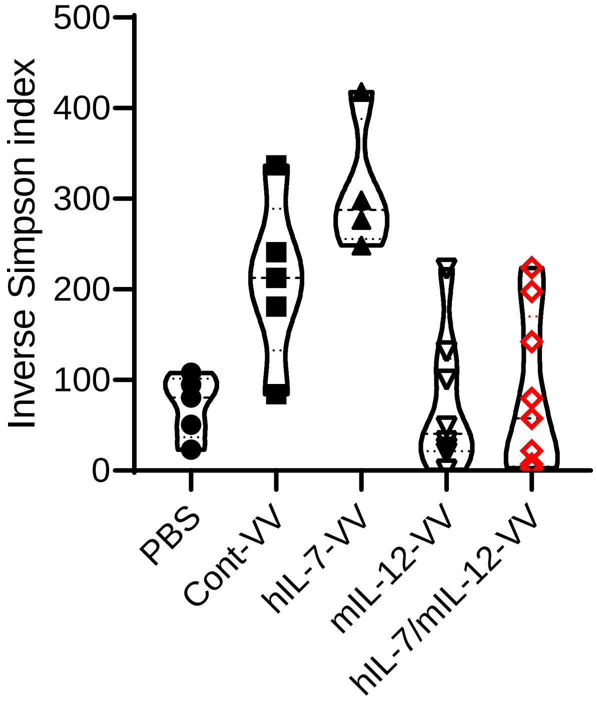

Clonality index (inverse Simpson's index) of intratumoral CD4^{+} T cells in each treatment group, as described in Figure 2.

Supplementary Figure 7.

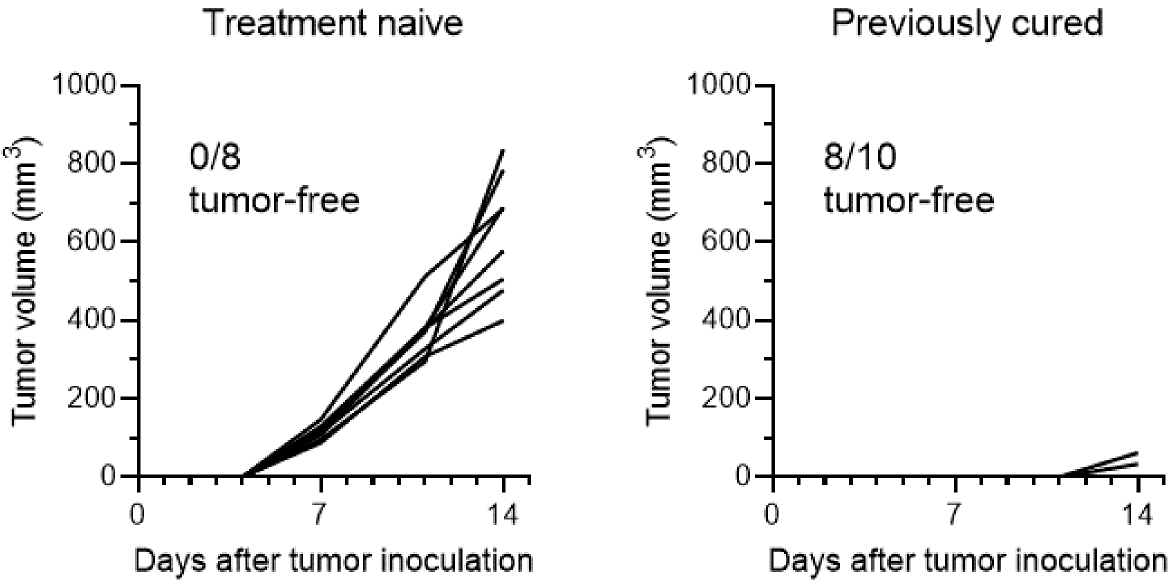

Rejection of rechallenged tumors in mice that had achieved complete tumor regression after treatment with hIL-7/mIL-12-VV. Mice that had achieved complete regression against LLC tumors after intratumoral treatment with hIL-7/mIL-12-VV and age-matched treatment naive mice were subcutaneously inoculated with LLC cells. Tumor growth in individual mice is shown.

Supplementary Table 1. Frequency of top 5 intratumoral CD4$^+$ T-cell clones in the spleen

Mouse	CDR3β sequence	% in tumor	% in spleen
PBS-1	CASSPLGYAEQFF	3.16	0.00
	CASSLSRGPNERLFF	3.13	0.00
	CASSLAGSGNTLYF	2.83	0.00
	CASSQPGQTEVFF	2.56	0.00
	CASSLPGGYEQYF	2.42	0.00
PBS-2	CASSHRGEQYF	4.08	0.00
	CASSLSRGGSPLYF	2.63	0.00
	CASSRTDNYAEQFF	2.61	0.00
	CTCSAPDNYEQYF	2.61	0.01
	CASSFFNQDTQYF	2.25	0.00
PBS-3	CASSDEGRAYEQYF	9.67	0.00
	CASSQENSGNTLYF	8.44	0.00
	CASSLDDQNTLYF	7.65	0.00
	CASRDRGGGQNTLYF	7.34	0.00
	CASSPDRDAEQFF	5.69	0.03
PBS-4	CASSPPGGWNTLYF	7.82	0.00
	CTCSATGTGNSDYTF	6.60	0.00
	CASSPQGPGNTLYF	3.58	0.00
	CASGVGGRDEQYF	2.19	0.00
	CASSLGQLSNERLFF	1.96	0.00
PBS-5	CASGEGLGGLAEQFF	8.22	0.21
	CASSLDISQNTLYF	2.34	0.00
	CASSLELGGPEQYF	2.20	0.00
	CSSSPSGGAYEQYF	2.13	0.58
	CASSQDPGGFYEQYF	1.55	0.00
Cont-VV-1	CTCSAGGSGNTLYF	9.43	0.00
	CASSPTGNTEVFF	1.25	0.00
	CASSQLNF	1.23	0.00
	CASSLWGGAGTGQLYF	1.09	0.02
	CASSQDPYNSPLYF	1.04	0.00
Cont-VV-2	CASSQEGLGGREQYF	1.93	0.00
	CASSISRSGNTLYF	1.77	0.16
	CASSFQGSNSDYTF	1.65	0.01
	CTCSAGRGSQNTLYF	1.55	0.03
	CASSLRDWGVAEQFF	1.50	0.00
Cont-VV-3	CASSLQGGRDTQYF	1.63	0.15
	CASSLQGGTEVFF	1.59	0.00
	CASSLEGDTLYF	1.50	0.10
	CASSQVNTEVFF	1.33	0.00
	CASSRRDRATEVFF	1.33	0.03
Cont-VV-4	CASSLTQEQYF	1.74	0.03
	CASGLDWGGDQDTQYF	1.06	0.00
	CASSLDWGSQNTLYF	0.95	0.00
	CASSPGQNTEVFF	0.90	0.00
	CASSPGTGGYEQYF	0.90	0.00
Cont-VV-5	CASSLESQNTLYF	2.72	0.03
	CASSLSGNTLYF	2.26	0.00
	CSSSQLWANTEVFF	1.43	0.00
	CASSIGTGAYEQYF	1.27	0.00
	CASSLDWGQDTQYF	1.18	0.00
hIL-7-VV-1	CASSLGQNTEVFF	1.81	0.01
	CASGDEAGQTNTEVFF	1.60	0.00
	CASSRQDQAPLF	1.43	0.00
	CASSRMEDTQYF	1.24	0.01
	CASSPTGDQDTQYF	1.00	0.00
hIL-7-VV-2	CASSPGTVNTGQLYF	1.37	0.00
	CASSRDWGSQNTLYF	1.19	0.01
	CTCSAVGGGAETLYF	1.07	0.19
	CASSPGPNTEVFF	1.03	0.00
	CASSRSNERLFF	0.80	0.00
hIL-7-VV-3	CASSLGTGGSDYTF	1.79	0.00
	CASGGGANTEVFF	1.68	0.00
	CASSLGQNTEVFF	1.30	0.00
	CASSHRAERLFF	1.30	0.00
	CASSQEGINTGQLYF	1.19	0.00
hIL-7-VV-4	CASGDAYITEVFF	2.19	0.00
	CASSLALWGYEQYF	1.11	0.00
	CASSQQGWEVFF	1.11	0.00
	CASSQEGTGNERLFF	1.07	0.06
	CSSSRQGSETLYF	1.04	0.00

Mouse	CDR3β sequence	% in tumor	% in spleen
mIL-12-VV-1	CTCSAGQGWEQYF	8.51	0.00
	CASSLEGGQDTQYF	7.60	0.18
	CTCSALGTSQDTQYF	6.25	0.00
	CASSDGGTEVFF	5.31	0.00
	CASSPDWASSYEQYF	4.98	0.00
mIL-12-VV-2	CASGDADRRGSDYTF	89.65	0.00
	WASGDADRRGSDYTF	0.48	0.00
	CASGDADRRGSDYTV	0.31	0.00
	CAGGDADRRGSDYTF	0.25	0.00
	CASGDVDRRGSDYTF	0.20	0.00
mIL-12-VV-3	CASSGQGNTEVFF	6.45	0.30
	CASSRDWGDTQYF	6.30	0.48
	CASSPMTGGEQYF	6.30	0.00
	CASSLETDNQDTQYF	5.80	0.00
	CASSRLGVQNTLYF	5.29	0.10
mIL-12-VV-4	CASSLEGQNTLYF	16.07	0.07
	CASGDAWGGYEQYF	7.79	0.00
	CGARETGDEQYF	5.39	0.04
	CASRTAERLFF	5.14	0.00
	CASSLEGGSNERLFF	4.78	0.03
hIL-7/mIL-12-VV-1	CASRVRGRDTQYF	6.53	0.00
	CASSQTYNYAEQFF	5.44	0.03
	CASSLEGGEDTQYF	3.22	0.00
	CASGDAGGALGEQYF	3.20	0.00
	CTCSADPGTGTEVFF	2.17	0.00
hIL-7/mIL-12-VV-2	CAWSLMRDTEVFF	43.62	0.00
	CASSLWGGAEQYF	43.07	0.00
	CASSLDTNTGQLYF	5.45	0.00
	CASSRWGGAEQYF	0.21	0.00
	CASALWGGAEQYF	0.19	0.00
hIL-7/mIL-12-VV-3	CASSFYVFF	49.60	0.00
	CASSDPGGTETLYF	31.61	0.03
	CTCSATGEGNTLYF	13.00	0.00
	CASSCYVFF	0.17	0.00
	CASSVYVFF	0.14	0.00
hIL-7/mIL-12-VV-4	CASSYSQNTLYF	13.90	0.03
	CASGGGGDNNQAPLF	10.96	0.00
	CASSLQGFYNSPLYF	3.25	0.01
	CAWSLGLGDQDTQYF	3.19	0.00
	CTCSADGTGDYEQYF	3.19	0.01
hIL-7/mIL-12-VV-5	CASSLPGQGGSQNTLYF	2.92	0.13
	CASSQPGQTNERLFF	1.77	0.00
	CSSSQGTGGYEQYF	1.30	0.03
	CASGDWGANTGQLYF	1.30	0.00
	CAWSLGIANSDYTF	1.29	0.01

Information including TRBV, TRBJ, and the number of reads is described in Supplementary Data File 2.
